# Supplementary material for: Dephosphorylation of the HIV-1 restriction factor SAMHD1 is mediated by PP2A-B55α holoenzymes during mitotic exit
Source: Nat Commun. 2018 Jun 8;9:2227. doi: 10.1038/s41467-018-04671-1 (PMC5993806; doi:10.1038/s41467-018-04671-1)
Supplement: Supplementary file 1 — Supplementary Information [file 41467_2018_4671_MOESM1_ESM.pdf]

Supplementary Information

Dephosphorylation of the HIV-1 restriction factor SAMHD1 is mediated by PP2A-B55α holoenzymes during mitotic exit

Schott et al.

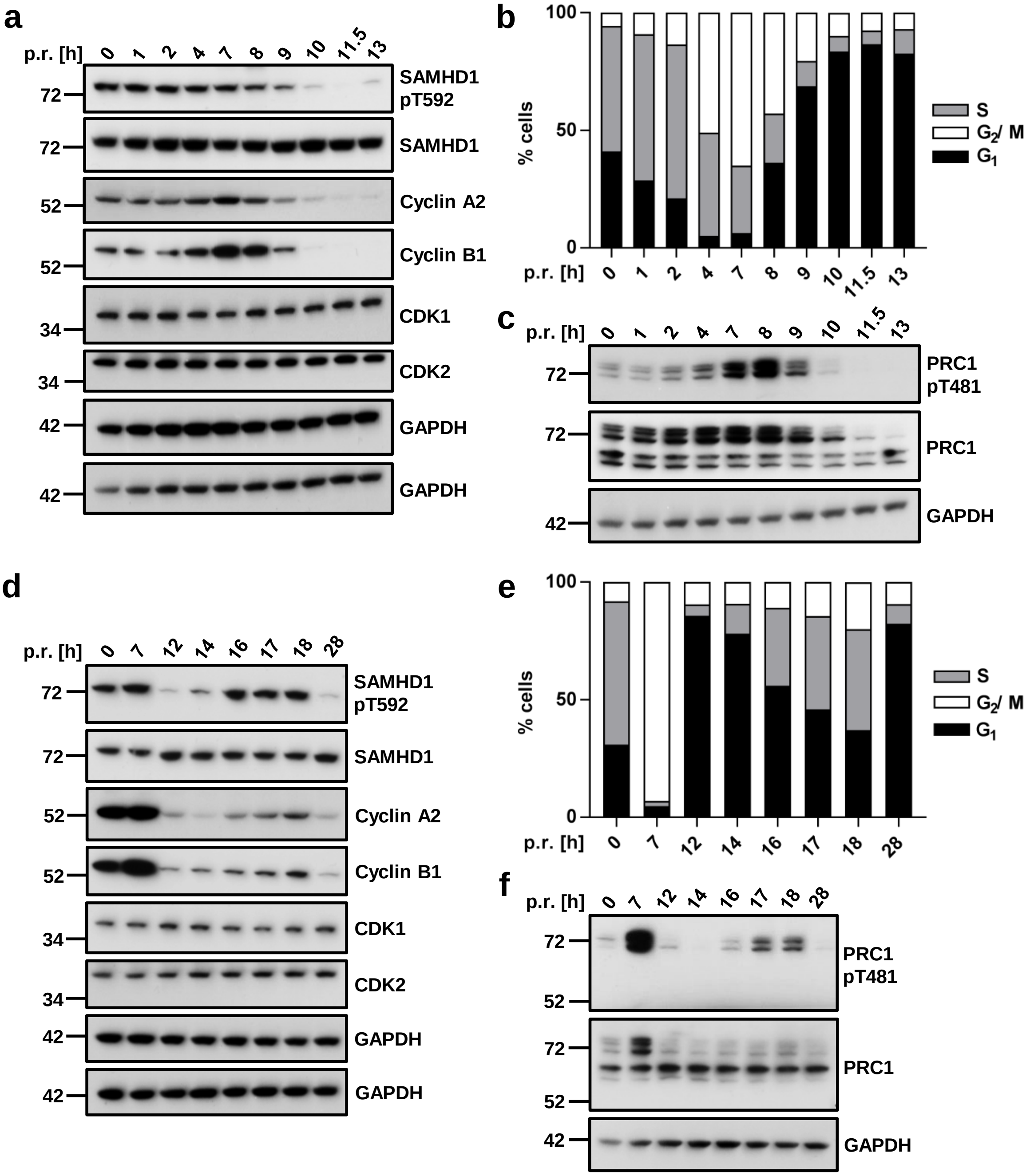

Supplementary Figure 1: SAMHD1 (de)phosphorylation at T592 is regulated in a cell cycle-dependent manner – related to Figure 1.

(a)-(c) Lower dATP concentrations upon G_1 entry are paralleled by SAMHD1 pT592 dephosphorylation. HeLa cells were arrested at the G_1/ S border using a double-thymidine block. After the 2nd release, synchronized cells were harvested at different time points post-release (p.r.). Respective samples were split for dNTP measurements (Fig. 1c), immunoblotting **(a)+(c)** and propidium iodide (PI) staining **(b)** to determine cell cycle-phases by flow cytometry. For immunoblotting, whole-cell lysates were analyzed using antibodies specific to the indicated proteins. Data shown are representative of two independent experiments.

(d)-(f) SAMHD1 is phosphorylated at T592 upon re-entry into S phase. HeLa cells were arrested at the G_1/ S border and harvested at different time points as described in Supplementary Fig. 1a. Respective samples were split for immunoblotting **(d)+(f)** and PI staining **(e)** to determine cell cycle-phases by flow cytometry. For immunoblotting, whole-cell lysates were analyzed using antibodies specific to the indicated proteins. Data shown are representative of two independent experiments.

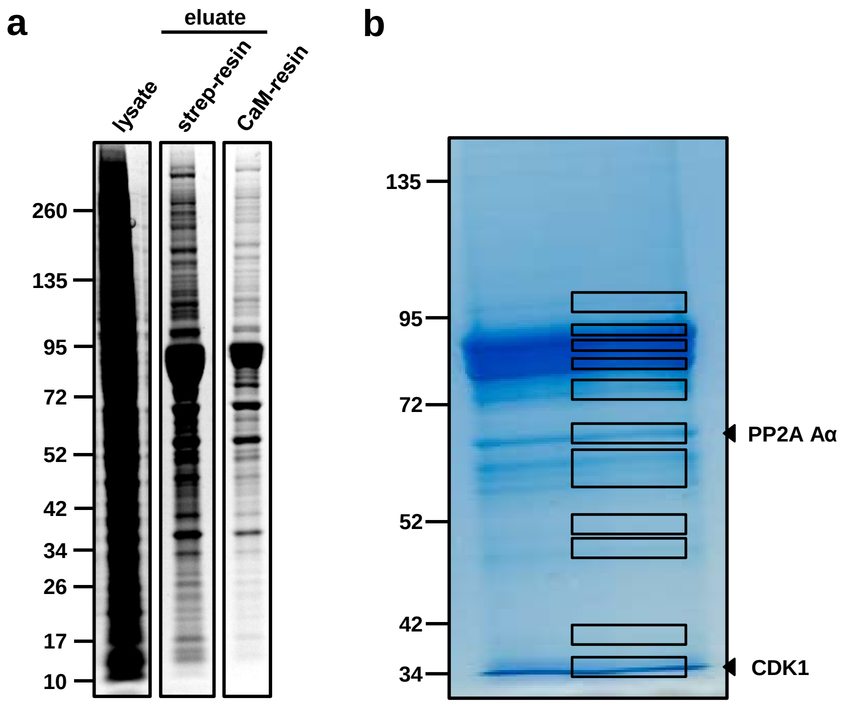

c

Identified protein	Acc. No.	MW	S	SC	E	P
Cyclin-dependent kinase 1 (CDK1)	P06493	34.1	190	17.8	4.9	4
Protein phosphatase 2A (PP2A) Aα subunit	P30153	65.3	171	17.0	2.5	7

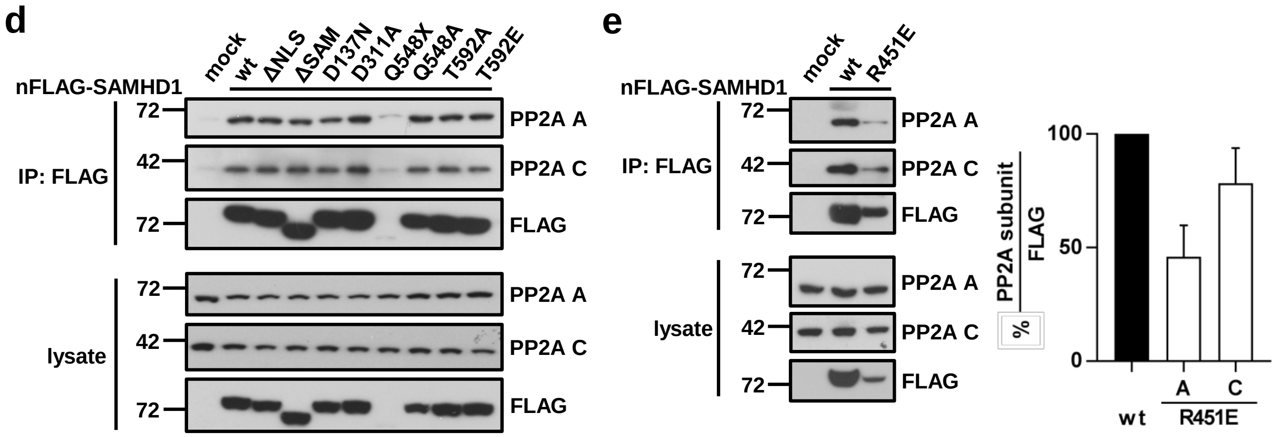

Supplementary Figure 2: Identification of PP2A Aα subunit and CDK1 as SAMHD1-interacting proteins in MS analysis (in-gel digestion of tandem affinity purified CBP-SBP-SAMHD1) – related to Figure 2.

(a) Samples of intermediate purification steps were separated by SDS-PAGE and stained with SYPRO Ruby.

(b) Purified protein complexes were eluted from the calmodulin (CaM)-resin and concentrated via TCA precipitation. 20 µg total protein were subjected to SDS-PAGE and stained with Coomassie Brilliant Blue G-250 overnight. After destaining, indicated protein bands were excised, treated with trypsin and analyzed using tandem MS (MS/MS).

(c) Identification of proteins relevant for T592 phosphorylation. Indicated are the protein name, UniProt accession number (Acc. No.), molecular weight (MW in [kDa]), protein score (S), sequence coverage (SC in [%]), average mass error (E in [ppm]) and number of identified unique peptides (P). The sequence coverage represents the percentage of a protein sequence represented by the peptides identified in the MS experiment.

(d) Interaction of endogenous PP2A A and C subunits with different SAMHD1 mutants. HEK293T cells were transfected with constructs expressing N-terminally FLAG-tagged SAMHD1 or various SAMHD1 point/ deletion mutants. Empty vector was transfected as a negative control. 48 h post-transfection, cells were harvested, lysed and CoIPs performed using anti-FLAG-coated agarose beads. Proteins were analyzed by immunoblotting using antibodies specific to the indicated proteins. Data shown are representative of three independent experiments.

(e) Interaction of PP2A A and C subunit with monomeric SAMHD1. HEK293T cells were transfected with constructs expressing N-terminally FLAG-tagged SAMHD1 or SAMHD1 R451E point mutant. Empty vector was transfected as a negative control. 48 h post-transfection, cells were harvested, lysed and CoIPs performed using anti-FLAG-coated agarose beads. Proteins were analyzed by immunoblotting using antibodies specific to the indicated proteins. To compare PP2A subunit binding to SAMHD1 wt and R451E mutant, the signals of each PP2A subunit were quantified and normalized to the respective FLAG signal (both from IP blot). Data shown are representative of three independent experiments; graph represents the mean ± SD of all experiments.

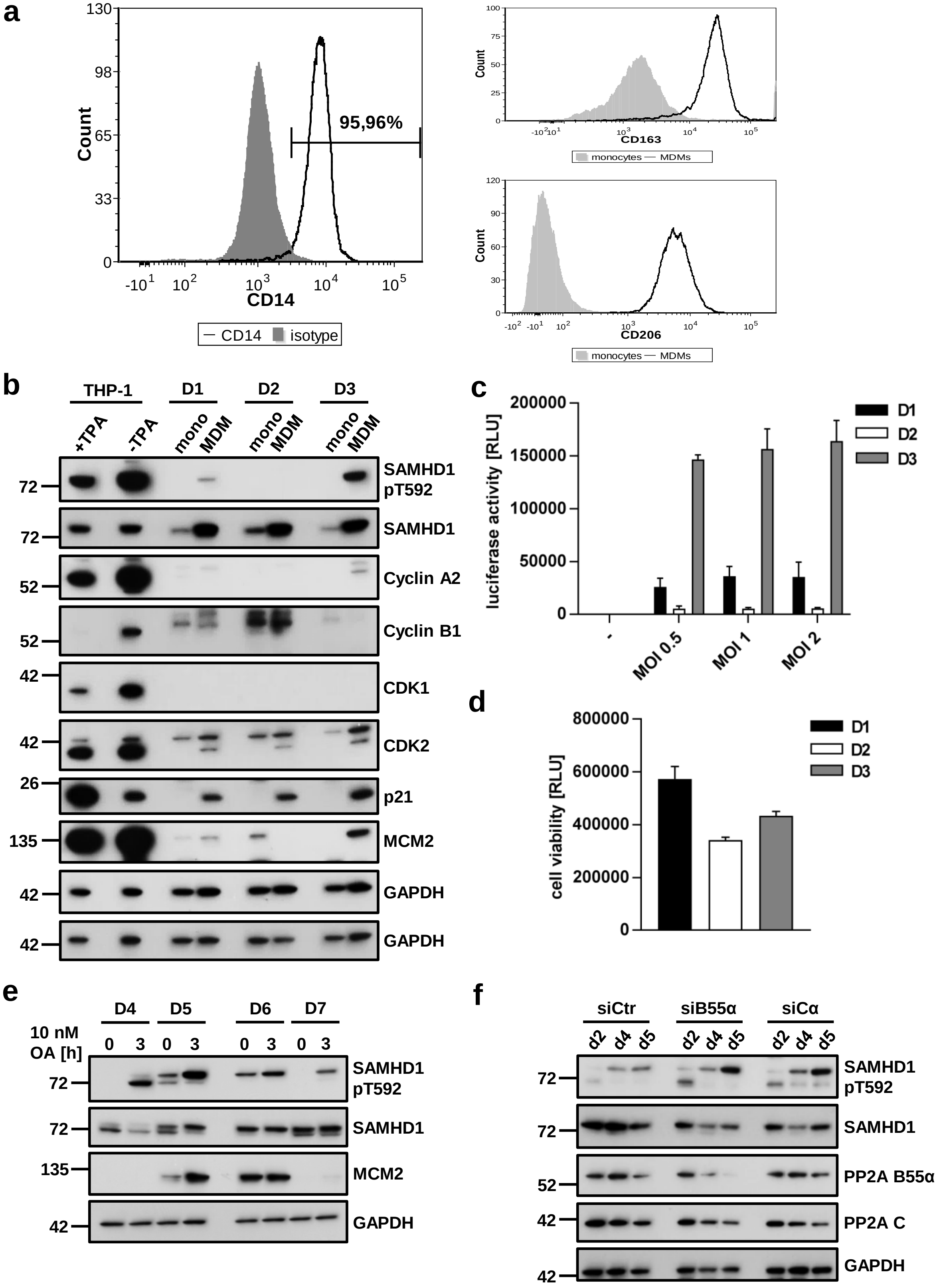
a
Count
CD14
95,96%
— CD14 isotype
Count
CD163
monocytes — MDMs
Count
CD206
monocytes — MDMs
b
THP-1
D1
D2
D3
+TPA
-TPA
mono
MDM
SAMHD1 pT592
SAMHD1
Cyclin A2
Cyclin B1
CDK1
CDK2
p21
MCM2
GAPDH
GAPDH
c
luciferase activity [RLU]
D1
D2
D3
-
MOI 0.5
MOI 1
MOI 2
d
cell viability [RLU]
D1
D2
D3
e
D4
D5
D6
D7
10 nM OA [h]
SAMHD1 pT592
SAMHD1
MCM2
GAPDH
f
siCtr
siB55α
siCα
d2
d4
d5
SAMHD1 pT592
SAMHD1
PP2A B55α
PP2A C
GAPDH

Supplementary Figure 3: Characterization of monocyte-derived macrophages (MDMs) – related to Figure 5.

(a) Assessment of MDM purity and differentiation status. MDMs were harvested after five days of differentiation and stained with antibodies (directly conjugated to fluorophores) specific to the indicated surface markers. Percentage of $CD14^{+}$ MDMs as well as increase of MDM-specific markers CD163/ CD206 for one donor are displayed. Data shown are representative of six donors analyzed.

(b) SAMHD1 T592 phosphorylation levels vary in MDMs between different donors. Monocytes were harvested directly after isolation, while MDMs from the respective donor were obtained after five days of differentiation. Whole-cell lysates were analyzed by immunoblotting using antibodies specific to the indicated proteins. Differentiated (+TPA) and cycling (-TPA) THP-1 cells are included as a control to illustrate the intensity of phosphorylation comparing various cell models. Data shown are representative of six donors analyzed.

(c)+(d) SAMHD1 T592 phosphorylation levels correlate with HIV-1 infection efficiency of MDMs from different donors (related to Supplementary Fig. 3b). MDMs were infected with VSV-G-pseudotyped HIV-1-luciferase reporter virus (MOI 0.5/ 1/ 2). After 24 h, infection efficiency was determined by measuring luciferase activity **(c)** and relative cell viability determined for each donor measured **(d)**. Graphs represent the mean ± SD of technical triplicates measured for each sample. Data shown are representative of six donors analyzed.

(e) Inhibition of PP2A by OA increases SAMHD1 phosphorylation at T592 in MDMs. MDMs were treated with the phosphatase inhibitor OA (10 nM) and harvested at different time points. Whole-cell lysates were analyzed by immunoblotting using antibodies specific to the indicated proteins. Data shown represent four donors analyzed.

(f) siRNA-mediated silencing of PP2A B55α and Cα subunits increases SAMHD1 T592 phosphorylation in MDMs over time. MDMs were transfected twice with control siRNA or a siRNA specifically targeting PP2A B55α or Cα subunit. At different time points post-transfection (2/ 4/ 5 days), cells were harvested and whole-cell lysates were analyzed by using antibodies specific to the indicated proteins. Data shown represent one of two donors analyzed.

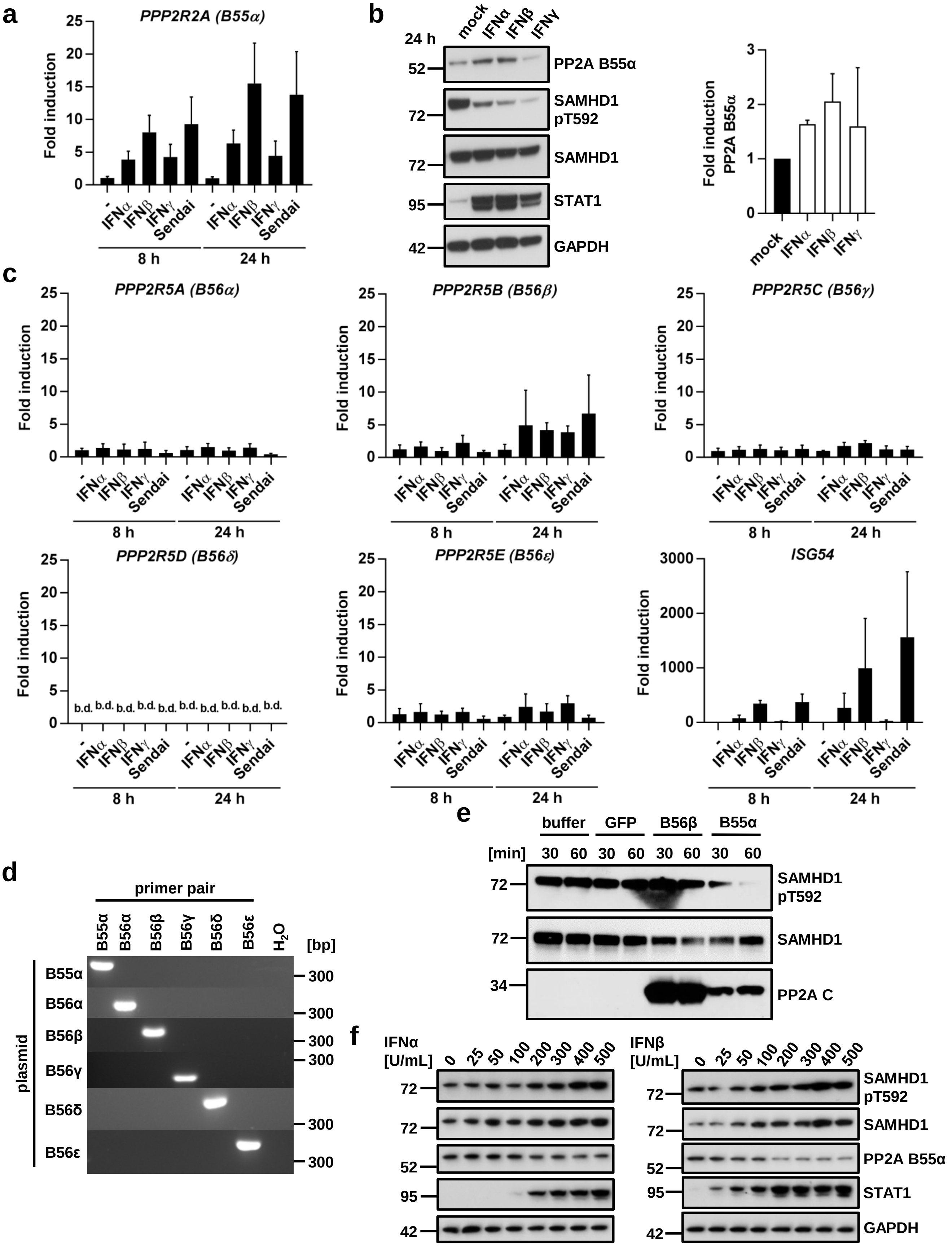
a
PPP2R2A (B55α)
Fold induction
- IFNα IFNβ IFNγ Sendai
8 h
24 h
b
24 h
mock IFNα IFNβ IFNγ
PP2A B55α
SAMHD1 pT592
SAMHD1
STAT1
GAPDH
52
72
72
95
42
Fold induction PP2A B55α
c
PPP2R5A (B56α)
PPP2R5B (B56β)
PPP2R5C (B56γ)
PPP2R5D (B56δ)
PPP2R5E (B56ε)
ISG54
b.d.
d
primer pair
B55α B56α B56β B56γ B56δ B56ε H2O
[bp]
plasmid
300
e
buffer GFP B56β B55α
[min] 30 60 30 60 30 60 30 60
SAMHD1 pT592
SAMHD1
PP2A C
34
f
IFNα [U/mL] 0 25 50 100 200 300 400 500
IFNβ [U/mL] 0 25 50 100 200 300 400 500
SAMHD1 pT592
SAMHD1
PP2A B55α
STAT1
GAPDH

Supplementary Figure 4: Regulation of specific PP2A B-type subunits by interferon (IFN).

(a) Type I and II IFNs induce mRNA upregulation of PP2A B55α subunit in MDMs. mRNA levels of PP2A B55α subunit in MDMs after treatment with different IFNs (IFNα, IFNβ, IFNγ; 1000 U/ mL each) for 8/ 24 h was quantified by RT-qPCR using specific primers (see Supplementary Fig. 4d). As a positive control for IFN-stimulated gene (ISG) induction, cells were infected with Sendai virus. Data were normalized to the reference gene *RPL13A*. Fold changes of mRNA levels to untreated samples (-) were calculated based on the mean of three donors, measured in three technical replicates each. Data shown represent the mean ± SD of the fold changes for three donors analyzed.

(b) SAMHD1 phosphorylation at T592 is reduced after IFN treatment in MDMs, while PP2A B55α protein expression is induced. Whole-cell lysates of MDMs, after treatment with different IFNs (IFNα, IFNβ, IFNγ; 1000 U/ mL each) for 24 h, were analyzed by immunoblotting using antibodies specific to the indicated proteins. For quantification, the signal of PP2A B55α subunit was normalized to GAPDH and compared to the mock-treated control (set as 1). Data shown represent one of three donors analyzed; graph represents the mean ± SD of all three donors analyzed.

(c) Type I and II IFNs do not or only slightly induce mRNA upregulation of PP2A B56 subunits in MDMs. mRNA levels of PP2A B56 subunits/ isoforms in MDMs after treatment with different IFNs (IFNα, IFNβ, IFNγ; 1000 U/ mL each) for 8/ 24 h were quantified by RT-qPCR using specific primers (see Supplementary Fig. 4d). As a positive control for IFN-stimulated gene (ISG) induction, cells were infected with Sendai virus. Data were normalized to the reference gene *RPL13A*. Fold changes of mRNA levels to untreated samples (-) were calculated based on the mean of three donors, measured in three technical replicates each (b.d. = below detection). Data shown represent the mean ± SD of the fold changes for three donors analyzed.

(d) Used qPCR primer pairs are isoform-specific for different PP2A B-type subunits. Each primer pair was tested against expression plasmids of all other subunits with PCR and analyzed on agarose gels to rule out cross-reactivity.

(e) Specific *in vitro* SAMHD1 pT592 dephosphorylation by PP2A-B55α, and not by PP2A-B56β trimers. The indicated PP2A trimers were retrieved from HEK293T cells as described in Fig. 5; anti-C immunoblotting indicates the amount of retrieved PP2A C subunit in these complexes. After *in vitro*-dephosphorylation by these PP2A trimers for the indicated times, SAMHD1 T592 phosphorylation was determined by immunoblotting.

(f) Protein levels of PP2A B55α subunit are regulated in a cell type-specific manner. HEK293T cells were incubated with increasing amounts of type I IFNs (left panel: IFNα, right panel: IFNβ) for 24 h. Whole-cell lysates were analyzed by immunoblotting using antibodies specific to the indicated proteins. Data shown are representative of two independent experiments (for each IFN).

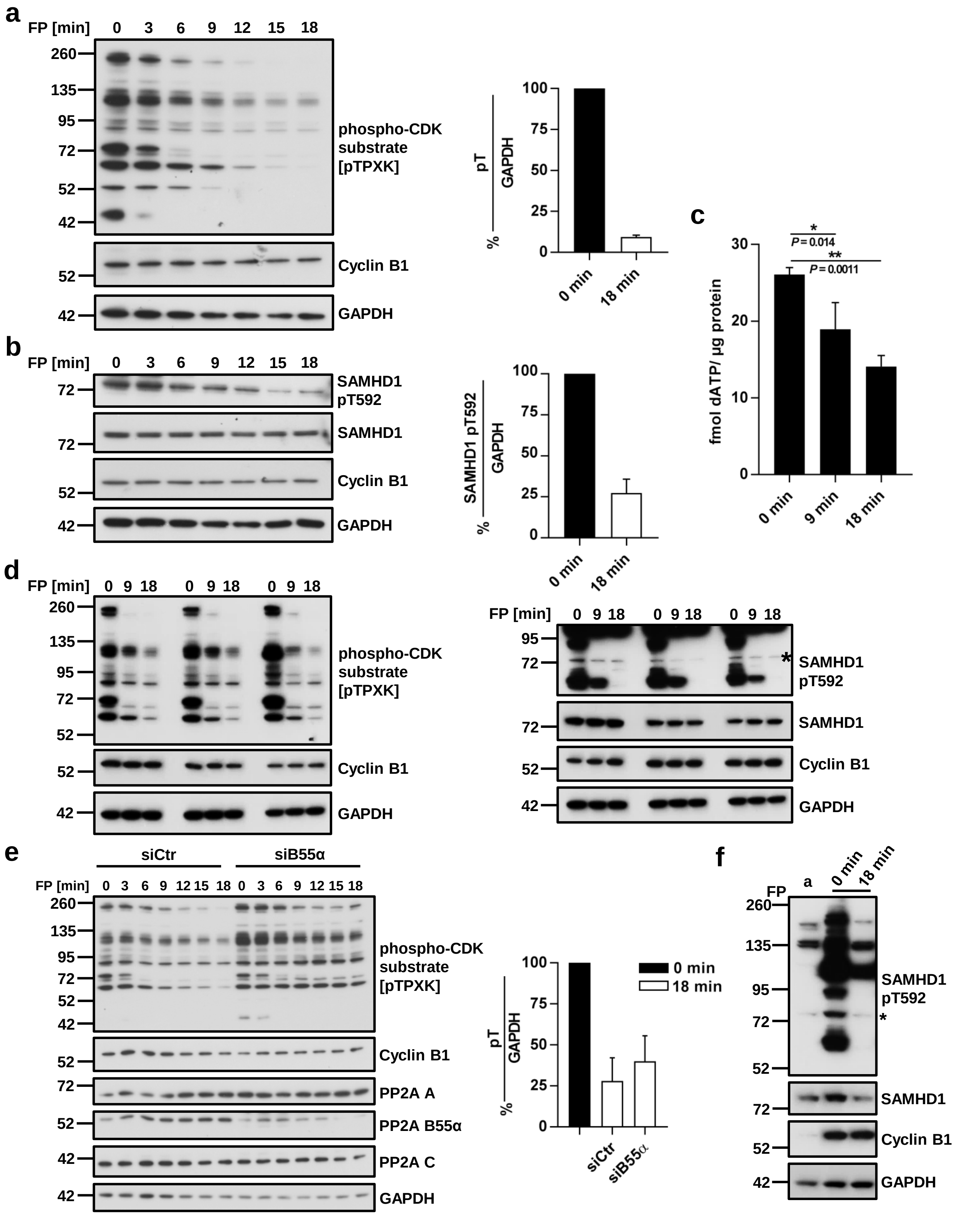

a
FP [min] 0 3 6 9 12 15 18
260
135
95
72
52
42
phospho-CDK substrate [pTPXK]
52
Cyclin B1
42
GAPDH
% pT / GAPDH
100
75
50
25
0
0 min
18 min
b
FP [min] 0 3 6 9 12 15 18
72
SAMHD1 pT592
72
SAMHD1
52
Cyclin B1
42
GAPDH
% SAMHD1 pT592 / GAPDH
100
75
50
25
0
0 min
18 min
c
*
P = 0.014
**
P = 0.0011
fmol dATP/ µg protein
30
20
10
0
0 min
9 min
18 min
d
FP [min] 0 9 18 0 9 18 0 9 18
260
135
95
72
52
phospho-CDK substrate [pTPXK]
52
Cyclin B1
42
GAPDH
FP [min] 0 9 18 0 9 18 0 9 18
95
72
*
SAMHD1 pT592
72
SAMHD1
52
Cyclin B1
42
GAPDH
e
siCtr
siB55α
FP [min] 0 3 6 9 12 15 18 0 3 6 9 12 15 18
260
135
95
72
52
42
phospho-CDK substrate [pTPXK]
52
Cyclin B1
72
PP2A A
52
PP2A B55α
42
PP2A C
42
GAPDH
% pT / GAPDH
100
75
50
25
0
0 min
18 min
siCtr
siB55α
f
FP
a
0 min
18 min
260
135
95
72
52
SAMHD1 pT592
*
72
SAMHD1
52
Cyclin B1
42
GAPDH

Supplementary Figure 5: SAMHD1 is dephosphorylated at T592 during mitotic exit - related to Figure 6.

(a)+(b) SAMHD1 is dephosphorylated at T592, paralleling overall CDK substrate dephosphorylation, during mitotic exit. HeLa cells were arrested in mitosis using nocodazole and mitotic exit was induced chemically by adding flavopiridol (FP) in the presence of MG-132. Cells were harvested at the indicated time points over a period of 18 min. Whole-cell lysates were analyzed by immunoblotting using antibodies specific to the indicated proteins. For quantification, the signal of overall CDK substrate phosphorylation pT (= whole lane) **(a)** or phosphorylated SAMHD1 (= SAMHD1 pT592) **(b)** was normalized to GAPDH. Level of normalized pT **(a)** or SAMHD1 pT592 **(b)** was determined within 18 min. Immunoblot data shown are representative of three independent experiments, while the quantification graphs represent the mean ± SD of all three experiments.

(c)+(d) dATP levels decrease during mitotic exit, paralleling SAMHD1 pT592 dephosphorylation. HeLa cells were arrested in mitosis using nocodazole and mitotic exit was induced chemically by adding flavopiridol (FP) in the presence of MG-132. Cells were harvested at the indicated time points over a period of 18 min. Respective samples were split for dATP measurement **(c)** and immunoblotting **(d)**. **(c)** dATP was quantified by single nucleotide incorporation assay and normalized by protein content of the corresponding lysate. Statistical significance was determined using a one-way ANOVA with multiple comparisons according to Dunnett (ns: $p \geq 0.05$; *: $p < 0.05$; **: $p < 0.01$; ***: $p < 0.001$). Data shown represent the mean ± SD of three independent experiments. **(d)** Whole-cell lysates were analyzed using antibodies specific to the indicated proteins (left panel: overall CDK substrate phosphorylation pT; right panel: SAMHD1 pT592). The signal of phosphorylated SAMHD1 (signal marked with asterisks) was compared to SAMHD1 pT592 signal in non-synchronized cells. Data shown comprise all three independent experiments.

(e) Knock-down of PP2A B55α subunit leads to impaired and delayed dephosphorylation of CDK substrates during mitotic exit (related to Fig. 6). HeLa cells were either transfected with control siRNA or simultaneously with a single siRNA targeting PP2A B55α subunit. Transfected cells were then arrested in mitosis using nocodazole and mitotic exit was induced chemically by adding flavopiridol (FP) in the presence of MG-132. Cells were harvested at the indicated time points over a period of 18 min. Whole-cell lysates were analyzed by immunoblotting using antibodies specific to the indicated proteins. For quantification, the signal of overall CDK substrate phosphorylation pT (= whole lane) was normalized to GAPDH and level of pT (within 18 min) compared between siCtr- and siB55α-transfected cells. Immunoblot data shown are representative of two independent experiments, while the quantification graphs represent the mean ± SD of both experiments.

(f) Signal of SAMHD1 pT592 in mitotic HeLa cells. HeLa cells were arrested in G_2/ M phase using nocodazole and mitotic exit was induced chemically by adding flavopiridol (FP) in the presence of MG-132. Cells were harvested at the indicated time points over a period of 18 min. Whole-cell lysates were analyzed by immunoblotting using antibodies specific to the indicated proteins. Phosphorylated SAMHD1 (= SAMHD1 pT592; signal marked with asterisks) was identified by comparing mitotic (= 0/ 18 min) to asynchronously (= a) growing cells and correlating the SAMHD1 pT592-band with total SAMDH1. Data shown are representative of three independent experiments.

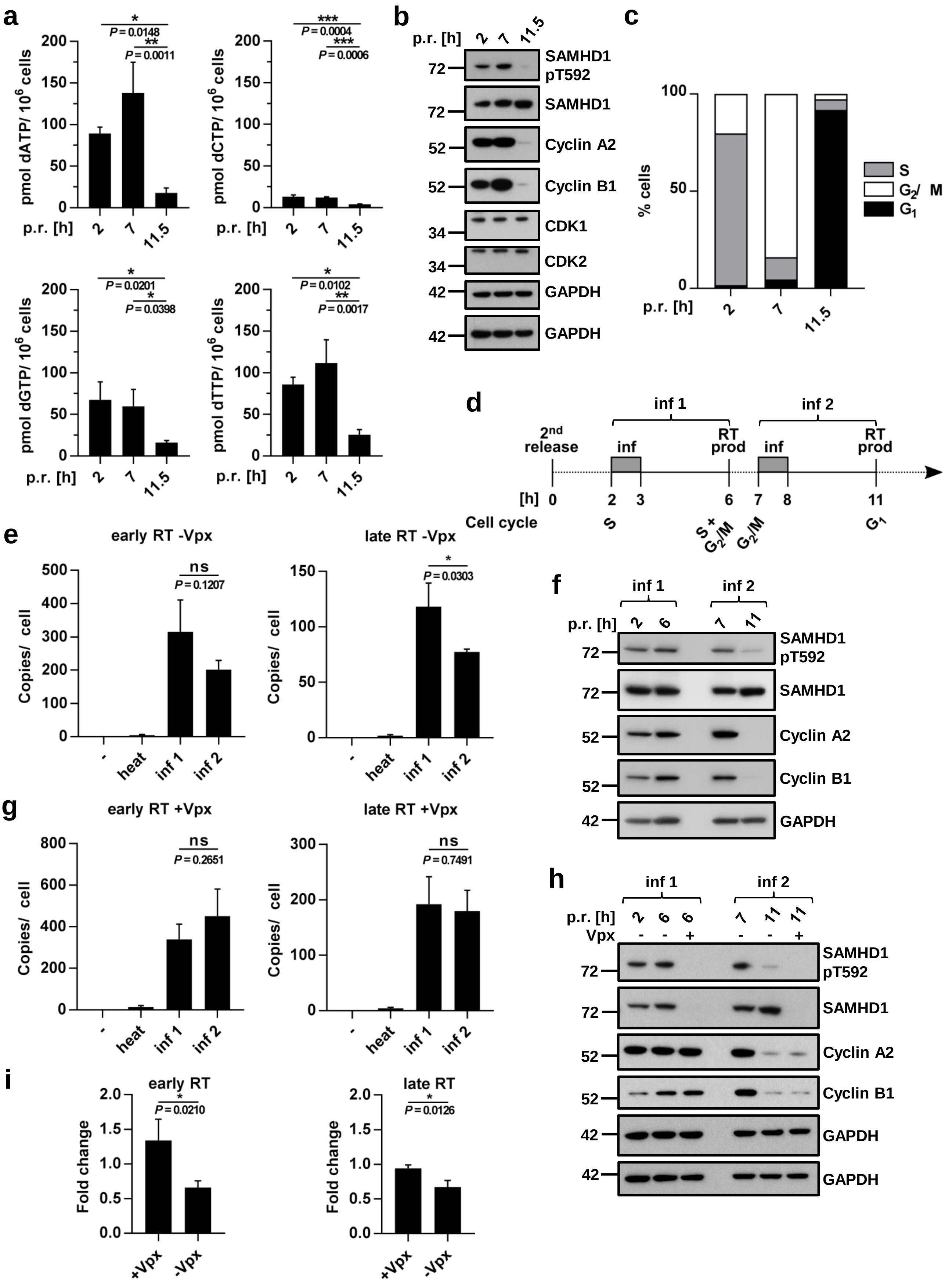

a
pmol dATP/ 10^6 cells
pmol dCTP/ 10^6 cells
pmol dGTP/ 10^6 cells
pmol dTTP/ 10^6 cells
p.r. [h]
2
7
11.5
P = 0.0148
P = 0.0011
P = 0.0004
P = 0.0006
P = 0.0201
P = 0.0398
P = 0.0102
P = 0.0017
b
SAMHD1 pT592
SAMHD1
Cyclin A2
Cyclin B1
CDK1
CDK2
GAPDH
c
% cells
S
G2/ M
G1
d
inf 1
inf 2
2nd release
inf
RT prod
[h]
Cell cycle
S
S + G2/M
G2/M
G1
e
early RT -Vpx
late RT -Vpx
Copies/ cell
ns
P = 0.1207
P = 0.0303
heat
inf 1
inf 2
f
g
early RT +Vpx
late RT +Vpx
P = 0.2651
P = 0.7491
h
Vpx
i
early RT
late RT
Fold change
P = 0.0210
P = 0.0126
+Vpx
-Vpx

Supplementary Figure 6: SAMHD1 dephosphorylation at T592 upon G_1 entry correlates with a decrease in HIV-1 RT products in cycling HeLa cells.

(a)-(c) G_1 entry results in lower concentration of all four dNTPs compared to S and G_2/ M phase in HeLa cells. HeLa cells were arrested at the G_1/ S border using a double-thymidine block. After the 2nd release, synchronized cells were harvested at different time points post-release (p.r.). Respective samples were split for dNTP measurements (dATP, dCTP, dGTP and dTTP) **(a)**, immunoblotting **(b)** and PI staining **(c)** to determine cell cycle-phases by flow cytometry. **(a)** dNTP amounts per million cells were quantified by single nucleotide incorporation assay. Statistical significance was determined using an ordinary one-way ANOVA with multiple comparisons according to Sidak (ns: $p \geq 0.05$; *: $p < 0.05$; **: $p < 0.01$; ***: $p < 0.001$). Data shown represent the mean ± SD of three independent experiments. **(b)** For immunoblotting, whole-cell lysates were analyzed using antibodies specific to the indicated proteins. Data shown in (b)+(c) are representative of three independent experiments.

(d) Schematic of infection experiment in synchronized HeLa cells. Cells were arrested at the G_1/ S border using a double-thymidine block. After the 2nd release, cells were infected with VSV-G-pseudotyped HIV-1 reporter virus (-/+ Vpx) at different time points (2/ 7 h p.r.) for 1 h. Total DNA for RT product measurements was harvested 4 h post-infection (6/ 11 h p.r.). Importantly, the time of infection was chosen in such a way that RT onset (3 h post-infection) would coincide with specific cell cycle-phases (2-6 h: S to G_2/ M = inf 1; 7-11 h: G_2/ M to G_1 = inf 2).

(e)+(f) Decrease in HIV-1 RT products in G_1 phase **(e)** correlates with SAMHD1 dephosphorylation at T592 **(f)**. **(e)** Synchronized HeLa cells were infected with VSV-G-pseudotyped HIV-1-luciferase reporter virus (MOI 3) or heat-inactivated virus. 4 h post-infection, total DNA was collected and the amount of early and late RT products determined by qPCR. Each sample was measured in technical triplicates. Statistical significance was determined using an unpaired, two-tailed Student's t-test (ns: $p \geq 0.05$; *: $p < 0.05$; **: $p < 0.01$; ***: $p < 0.001$). Data shown represent the mean ± SD of three independent experiments. **(f)** For immunoblotting, synchronized HeLa cells were harvested at time of infection (2/ 7 h p.r.) and DNA harvest (6/ 11 h p.r.). Whole-cell lysates were analyzed using antibodies specific to the indicated proteins. Data shown are representative of three independent experiments.

(g)+(h) No reduction of HIV-1 RT products in G_1 phase **(g)** after Vpx-induced SAMHD1 degradation **(h)**. **(g)** Synchronized HeLa cells were infected with VSV-G-pseudotyped HIV-1-luciferase reporter virus, which carried Vpx due to a mutation in p6, or heat-inactivated virus (MOI 1.5; virus amount in (g) and (e) was chosen to result in comparable copies/ cell between experiment (g) and (e)). 4 h post-infection, total DNA was collected and the amount of early and late RT products determined by qPCR. Each sample was measured in technical triplicates. Statistical significance was determined using an unpaired, two-tailed Student's t-test (ns: $p \geq 0.05$; *: $p < 0.05$; **: $p < 0.01$; ***: $p < 0.001$). Data shown represent the mean ± SD of three independent experiments. **(h)** For immunoblotting, synchronized HeLa cells were harvested at time of infection (2/ 7 h p.r.) and DNA harvest (6/ 11 h p.r.). Whole-cell lysates were analyzed using antibodies specific to the indicated proteins. Data shown are representative of three independent experiments.

(i) Significant reduction of HIV-1 RT products in G_1 phase is dependent on the absence of Vpx, consequently, presence of SAMHD1 (related to Supplementary Fig. 6d-h). Fold changes (= inf 2/ inf 1) in early and late RT products observed in Supplementary Fig. 6e, g after infection with VSV-G-pseudotyped HIV-1 reporter virus in presence and absence of Vpx were calculated (+/- Vpx). Statistical significance was determined using an unpaired, two-tailed Student's t-test (ns: $p \geq 0.05$; *: $p < 0.05$; **: $p < 0.01$; ***: $p < 0.001$). Data shown represent the mean fold change ± SD of three independent experiments.

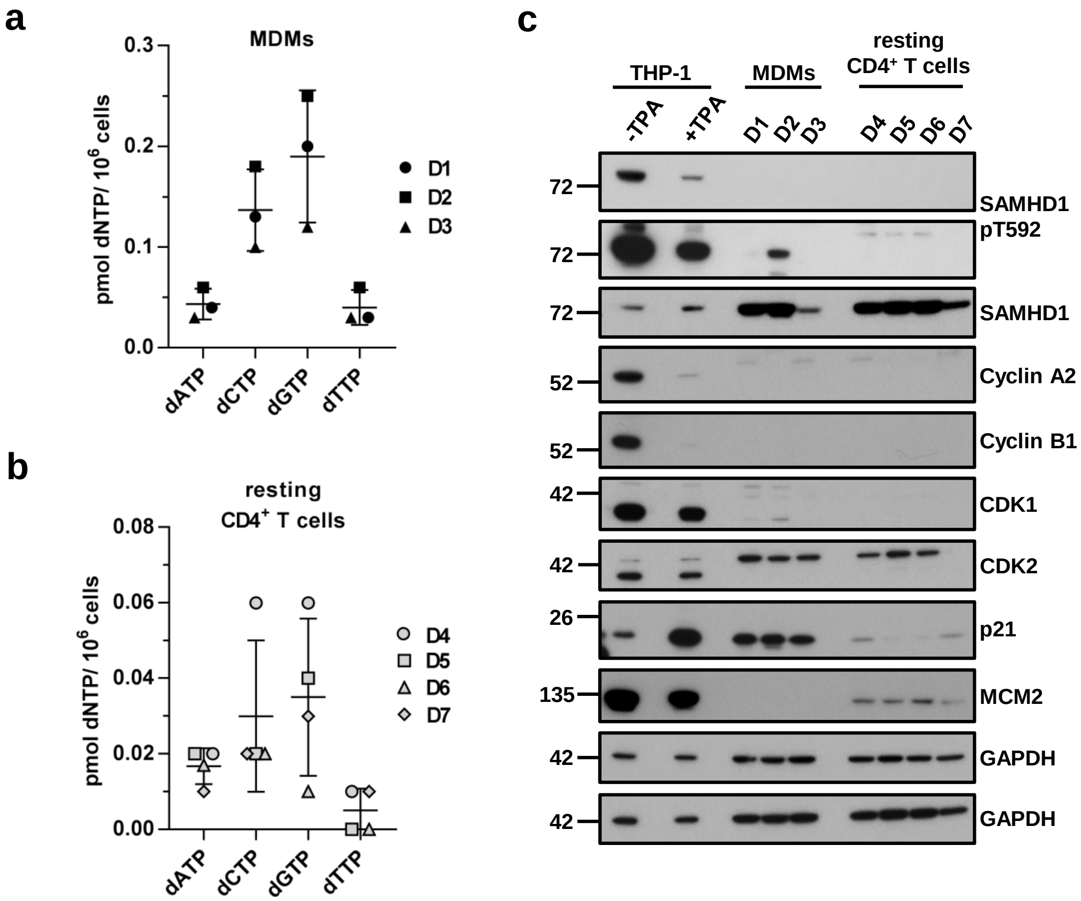

d

	Cell volume [µm³]	dATP [µM]	dATP Log [µM]	dCTP [µM]	dCTP Log [µM]	dGTP [µM]	dGTP Log [µM]	dTTP [µM]	dTTP Log [µM]
HeLa S	2349-3857	38.06-23.18	1.58-1.37	5.59-3.40	0.75-0.53	28.82-17.55	1.46-1.24	36.59-22.29	1.56-1.35
HeLa G_2/M		58.76-35.78	1.77-1.55	5.26-3.2	0.72-0.51	25.49-15.53	1.41-1.19	47.62-29.0	1.68-1.46
HeLa G_1		7.65-4.66	0.88-0.67	1.80-1.09	0.25-0.04	6.95-4.23	0.84-0.63	10.9-6.64	1.04-0.82
MDMs	2660	0.0163	-1.79	0.0514	-1.29	0.0714	-1.15	0.015	-1.82
resting CD4$^+$ T cells	186	0.0901	-1.05	0.161	-0.79	0.188	-0.73	0.0269	-1.57

Supplementary Figure 7: dNTP levels in human primary monocyte-derived macrophages (MDMs) and resting $CD4^+$ T cells.

MDMs from three different donors were obtained after five days of differentiation and analyzed in **(a)** and **(c)**; resting $CD4^+$ T cells from four different donors were harvested directly after isolation and analyzed in **(b)** and **(c)**.

(a)+(b) dNTP levels in human primary MDMs **(a)** or in human primary resting $CD4^+$ T cells **(b)**. dNTP amounts per million cells were quantified by single nucleotide incorporation assay. Data shown represent the mean ± SD of three donors (MDM) or four donors (resting $CD4^+$ T cells), respectively (each depicted by a specific symbol).

(c) SAMHD1 T592 phosphorylation levels in human primary MDMs and resting $CD4^+$ T cells. Whole-cell lysates were analyzed by immunoblotting using antibodies specific to the indicated proteins. Differentiated (+TPA) and cycling (-TPA) THP-1 cells are included as a control to illustrate the intensity of phosphorylation comparing various cell models. Data shown represent three (MDMs) or four (resting $CD4^+$ T cells) donors, respectively.

(d) Intracellular dNTP concentrations in different cell cycle-phases of synchronized HeLa cells compared to human primary MDMs/ resting $CD4^+$ T cells. dNTP amounts per million cells were quantified by single nucleotide incorporation assay (as described in Supplementary Fig. 6a and 7a, b). To calculate the average of intracellular dNTP concentrations, previously published cell volumes (HeLa cells: 2349-3857 μm^3; MDMs: 2660 μm^3; resting $CD4^+$ T cells: 186 μm^3) were used.

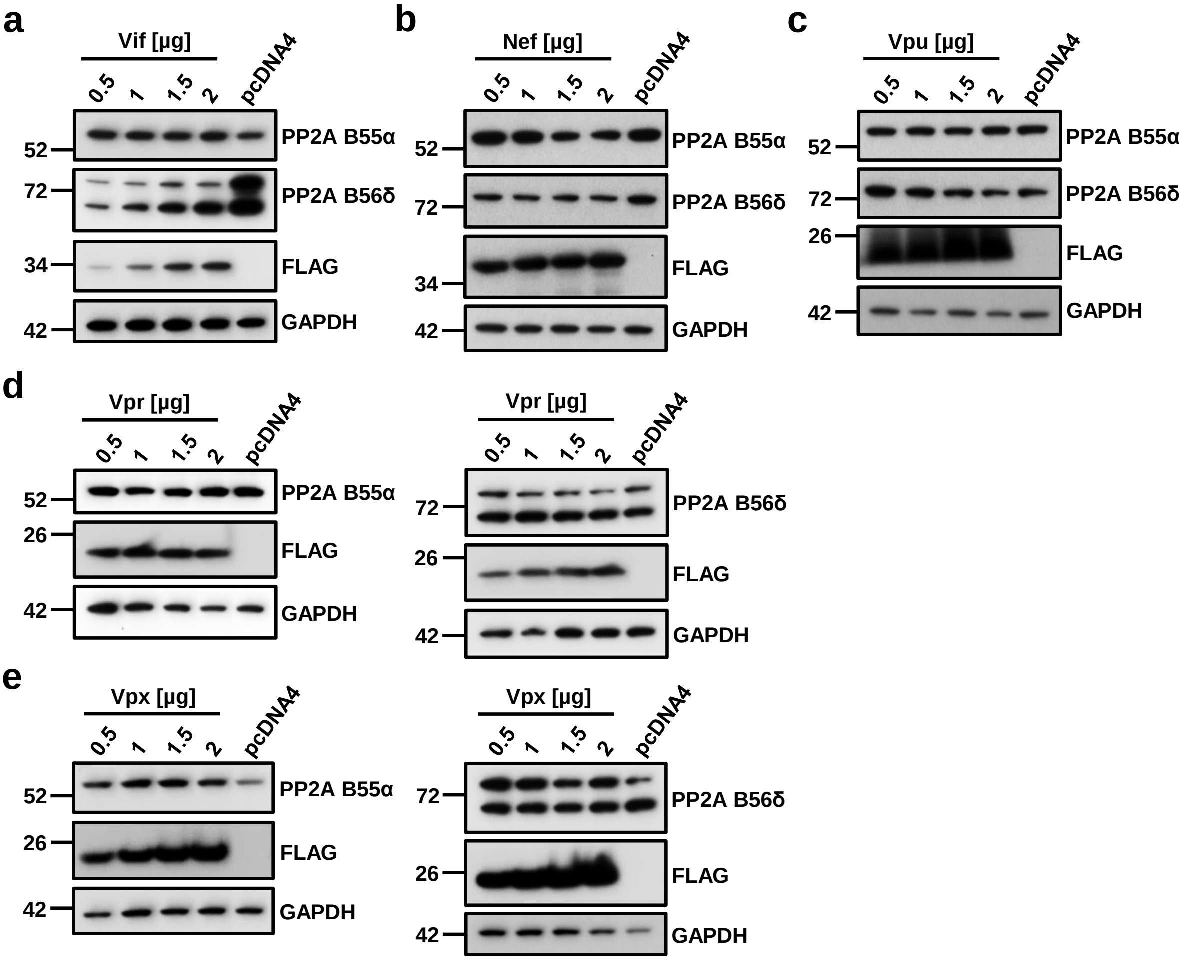

Supplementary Figure 8: Influence of lentiviral accessory proteins on PP2A B55α subunit expression – related to Figure 7.

HEK293T cells were transfected with increasing amounts of constructs expressing FLAG-tagged lentiviral accessory proteins Vif **(a)**, Nef **(b)**, Vpu **(c)**, Vpr **(d)**, Vpx **(e)** or empty vector as a negative control (= pcDNA4). 48 h post-transfection, cells were harvested and lysed. Proteins were analyzed by immunoblotting using antibodies specific to the indicated proteins. Data shown are representative of two or three independent experiments for Vif/ Vpr/ Vpx or Nef/ Vpu, respectively.

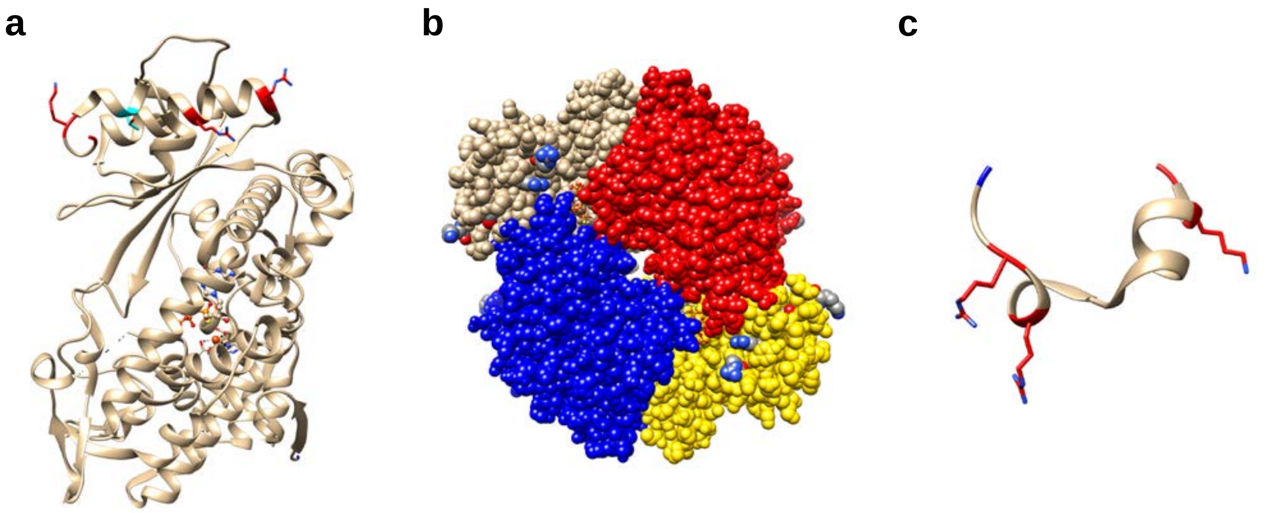

Supplementary Figure 9: Location of residues R559, R566, K596 and R609, R611, K622 within SAMHD1 structure determining the binding to PP2A-B55α holoenzymes.

(a) SAMHD1 monomer protein structure (PDB-ID: 4BZC, subunit C). Protein backbone is depicted in ribbon representation. Residues R559 (left), R566 (middle) and K596 (right) of the C-terminus are highlighted in red (nitrogen atoms of the side chains are colored blue). The phosphorylation-sensitive residue T592 is marked in cyan with the phosphorylated hydrogen group indicated in red. The enzymatic active site of the HD domain with the catalytic residues H167, H206, D207 and D311 are illustrated in complex with a magnesium ion (Mg^{2+}). The structure has bound the uncleavable nucleotide dGTPαS.

(b) SAMHD1 protein structure in tetramer formation (PDB-ID: 4BZC). Atoms of the protein subunits are depicted in sphere representation with colored subunits (A: tan; B: yellow; C: blue; D: red). Residues R559, R566 and K596 of the C-terminus are marked on all four subunits (atoms of the side chains are colored according to elements: C: grey, N: blue, O: red). The hydroxy group of the phosphorylation-sensitive residue T592 is visible in red. The structure has bound the nucleotide dGTPαS in all allosteric sites (depicted as ball-stick representation with atoms colored according to elements; C: grey, N: blue, O: red, P: orange, S: yellow). Representations of SAMHD1 structure **(a)+(b)** were generated using USCF Chimera based on published coordinates from Ji *et al.* (PDB-ID: 4BZC).

(c) C-terminus of SAMHD1 (residues N606-D624; PDB-ID: 4CC9). The protein backbone is shown in ribbon representation with residues R609 (left), R611 (middle) and K622 (right) highlighted in red (nitrogen atoms of the side chains are colored blue). The N- and C-terminus of the peptide have been colored blue and red, respectively. The structure was solved in complex with Vpx of sooty mangabey (Vpx_{sm}) and human DCAF1 (structures not shown). Representation of residues N606-D624 was generated using USCF Chimera based on published coordinates from Schwefel *et al.* (PDB-ID: 4CC9).

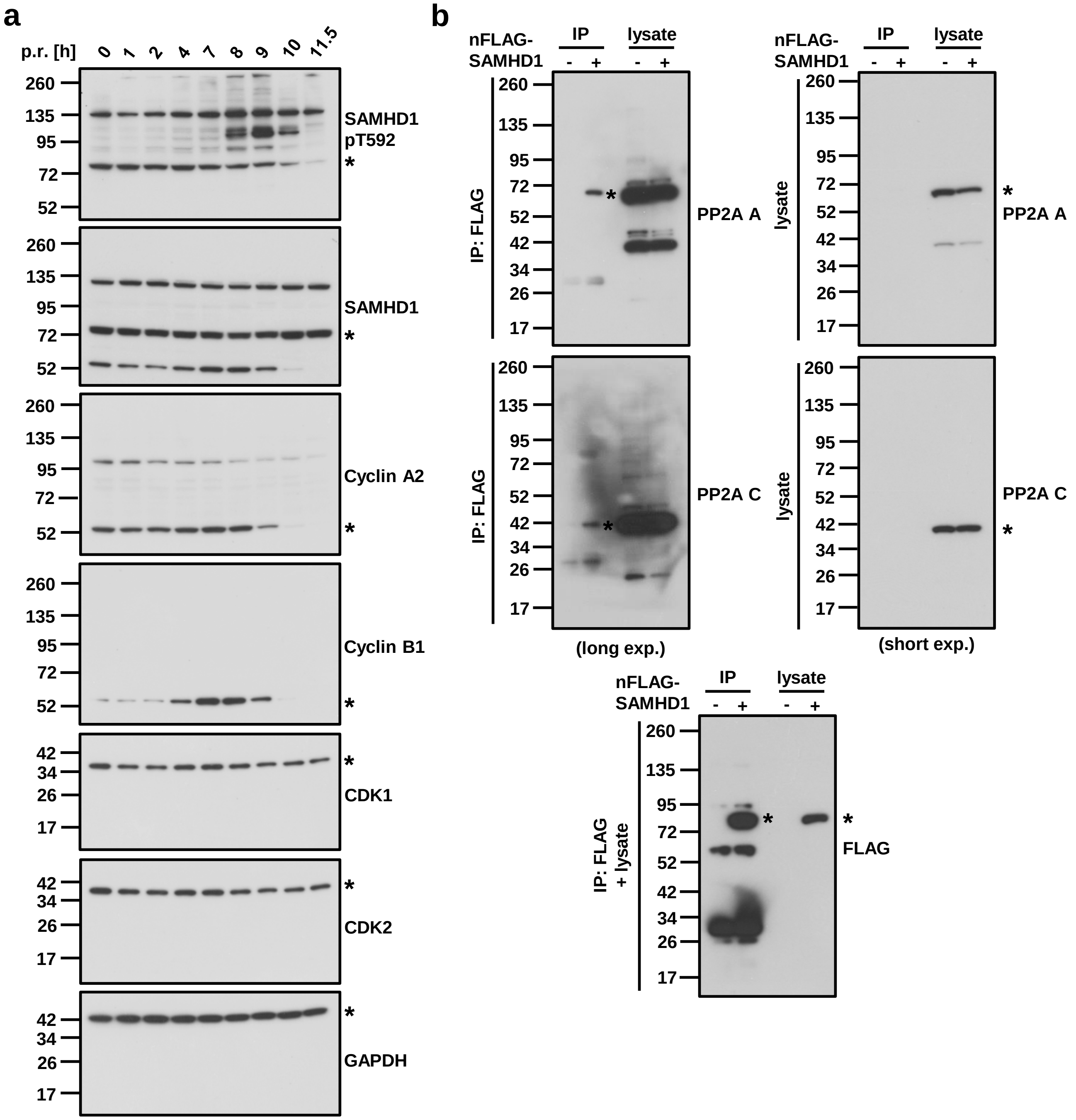

Supplementary Figure 10: Full scans of immunoblots – related to Figure 1a and Figure 2b.
(a) Full scan for SAMHD1 pT592, SAMHD1, cyclin A2, cyclin B1, CDK1, CDK2 and GAPDH immunoblots shown in Fig. 1a. Signals of respective proteins are marked with asterisks, as membranes were either probed consecutively with different antibodies (in some instances without stripping) or unspecific bands occurred. In some instances membranes were cut before probing.
(b) Full scan for PP2A A subunit, PP2A C subunit and FLAG immunoblots (IP + lysate) shown in Fig. 2b. Signals of respective proteins are marked with asterisks, as membranes were either probed consecutively with different antibodies (in some instances without stripping) or unspecific bands occurred.

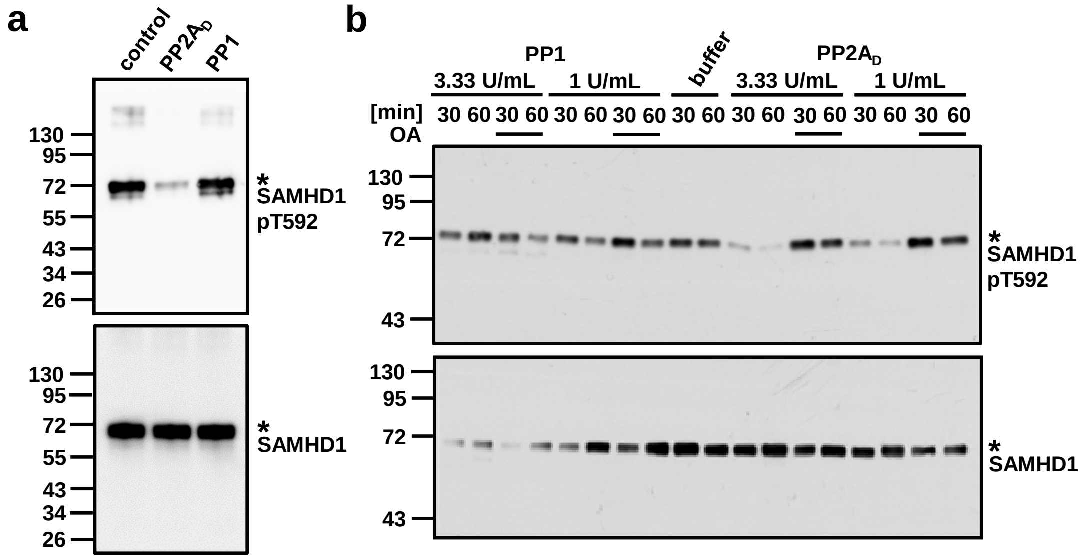

Supplementary Figure 11: Full scans of immunoblots – related to Figure 3b, c.
(a)+(b) Full scan for SAMHD1 pT592 and SAMHD1 immunoblots shown in **(a)** Fig. 3b and **(b)** Fig. 3c. Signals of respective proteins are marked with asterisks, as membranes were either probed consecutively with different antibodies (in some instances without stripping) or unspecific bands occurred.

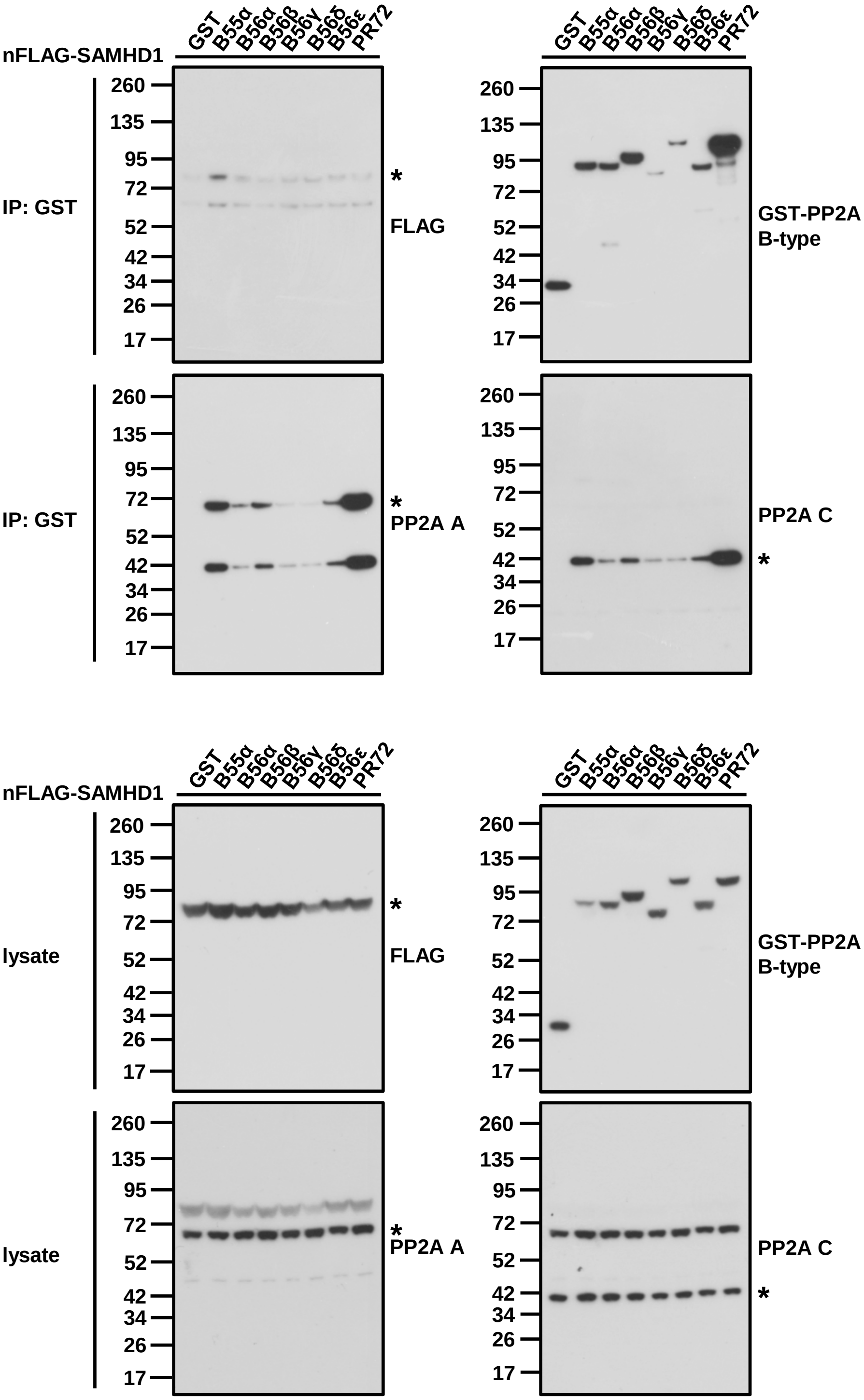

Supplementary Figure 12: Full scans of immunoblots – related to Figure 4a.
Full scan for FLAG, GST, PP2A A subunit and PP2A C subunit immunoblots (IP + lysate) shown in Fig. 4a. Signals of respective proteins are marked with asterisks, as membranes were either probed consecutively with different antibodies (in some instances without stripping) or unspecific bands occurred.

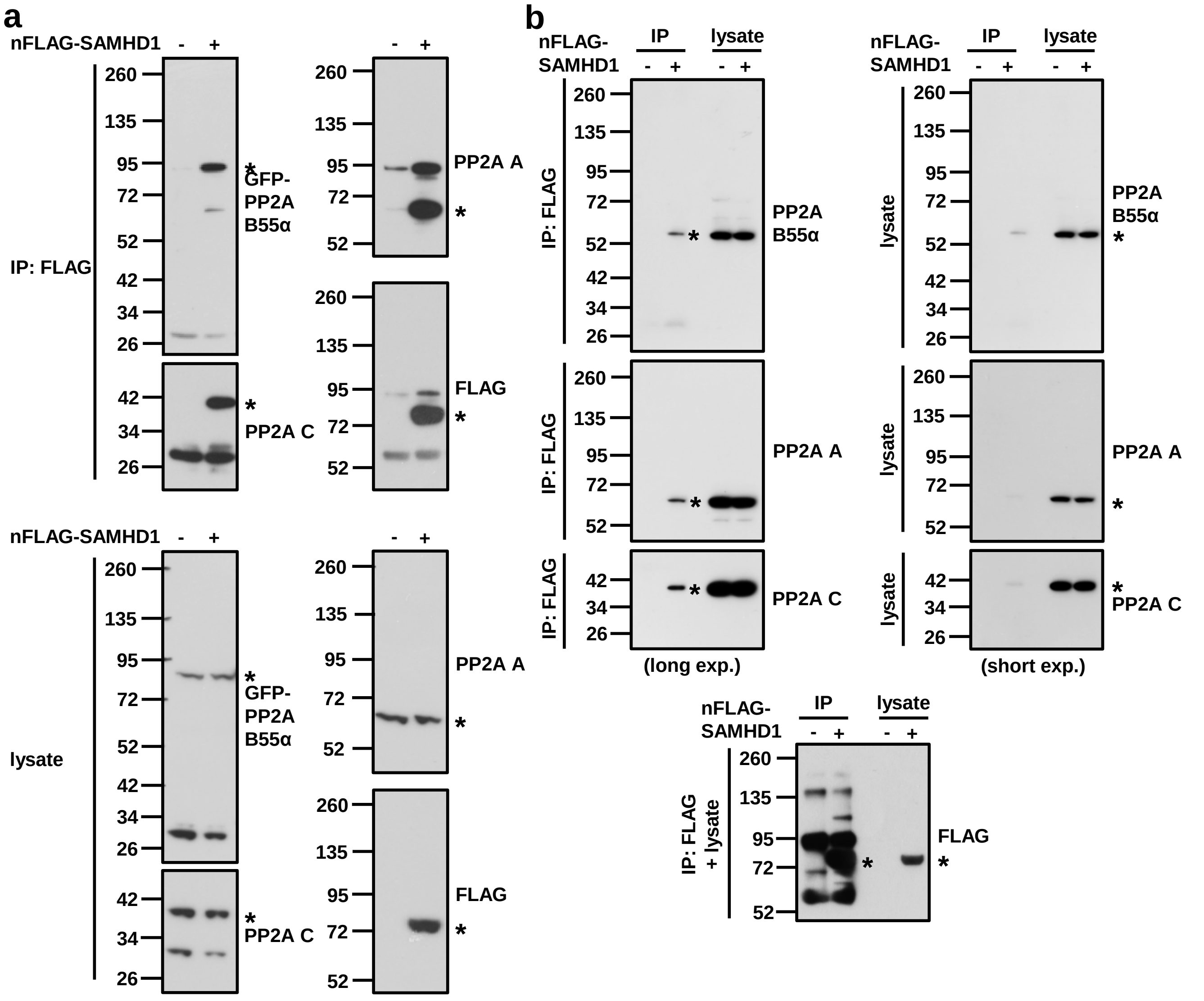

Supplementary Figure 13: Full scans of immunoblots – related to Figure 4b, c.

(a) Full scan for GFP, PP2A A subunit, PP2A C subunit and FLAG immunoblots (IP + lysate) shown in Fig. 4b. Signals of respective proteins are marked with asterisks, as membranes were either probed consecutively with different antibodies (in some instances without stripping) or unspecific bands occurred. In some instances membranes were cut before probing.

(b) Full scan for PP2A B55α subunit, PP2A A subunit, PP2A C subunit and FLAG immunoblots (IP + lysate) shown in Fig. 4c. Signals of respective proteins are marked with asterisks, as membranes were either probed consecutively with different antibodies (in some instances without stripping) or unspecific bands occurred. In some instances membranes were cut before probing.

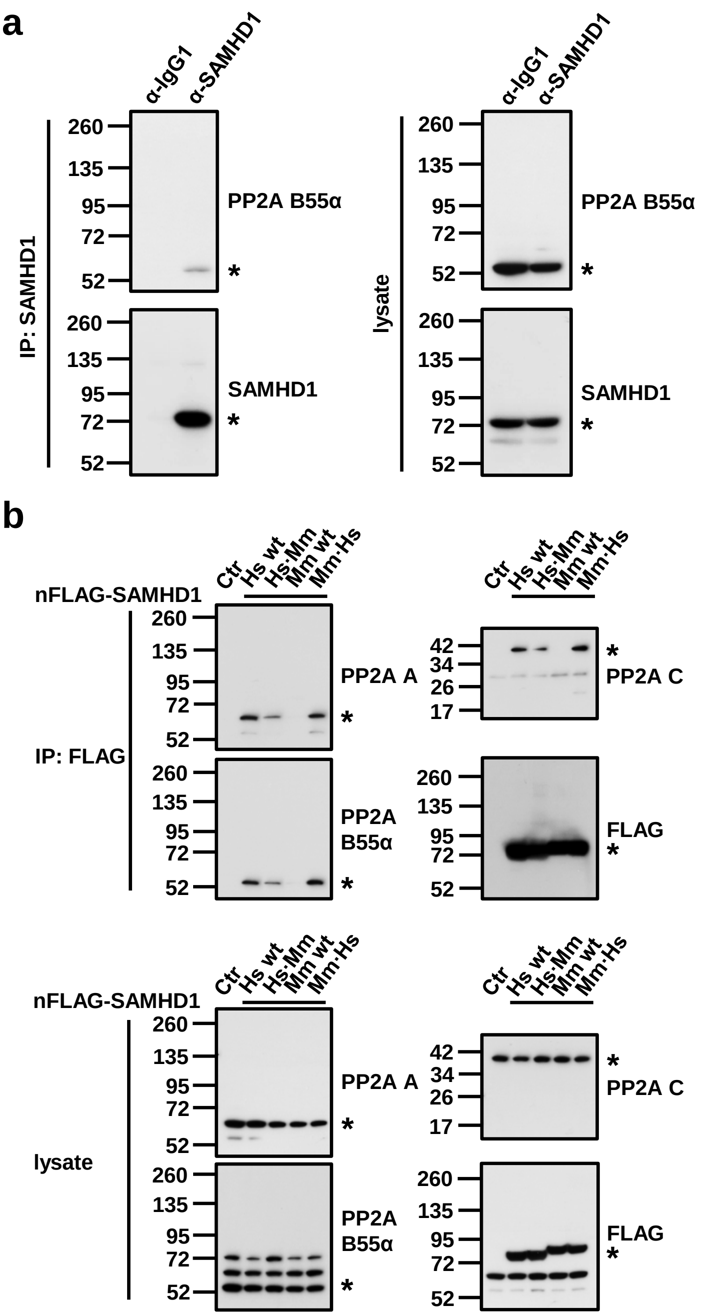

Supplementary Figure 14: Full scans of immunoblots – related to Figure 4d, f.
(a) Full scan for PP2A B55α subunit and SAMHD1 immunoblots (IP + lysate) shown in Fig. 4d. Signals of respective proteins are marked with asterisks, as membranes were either probed consecutively with different antibodies (in some instances without stripping) or unspecific bands occurred. In some instances membranes were cut before probing.
(b) Full scan for PP2A A subunit, PP2A B55α subunit, PP2A C subunit and FLAG immunoblots (IP + lysate) shown in Fig. 4f. Signals of respective proteins are marked with asterisks, as membranes were either probed consecutively with different antibodies (in some instances without stripping) or unspecific bands occurred. In some instances membranes were cut before probing.

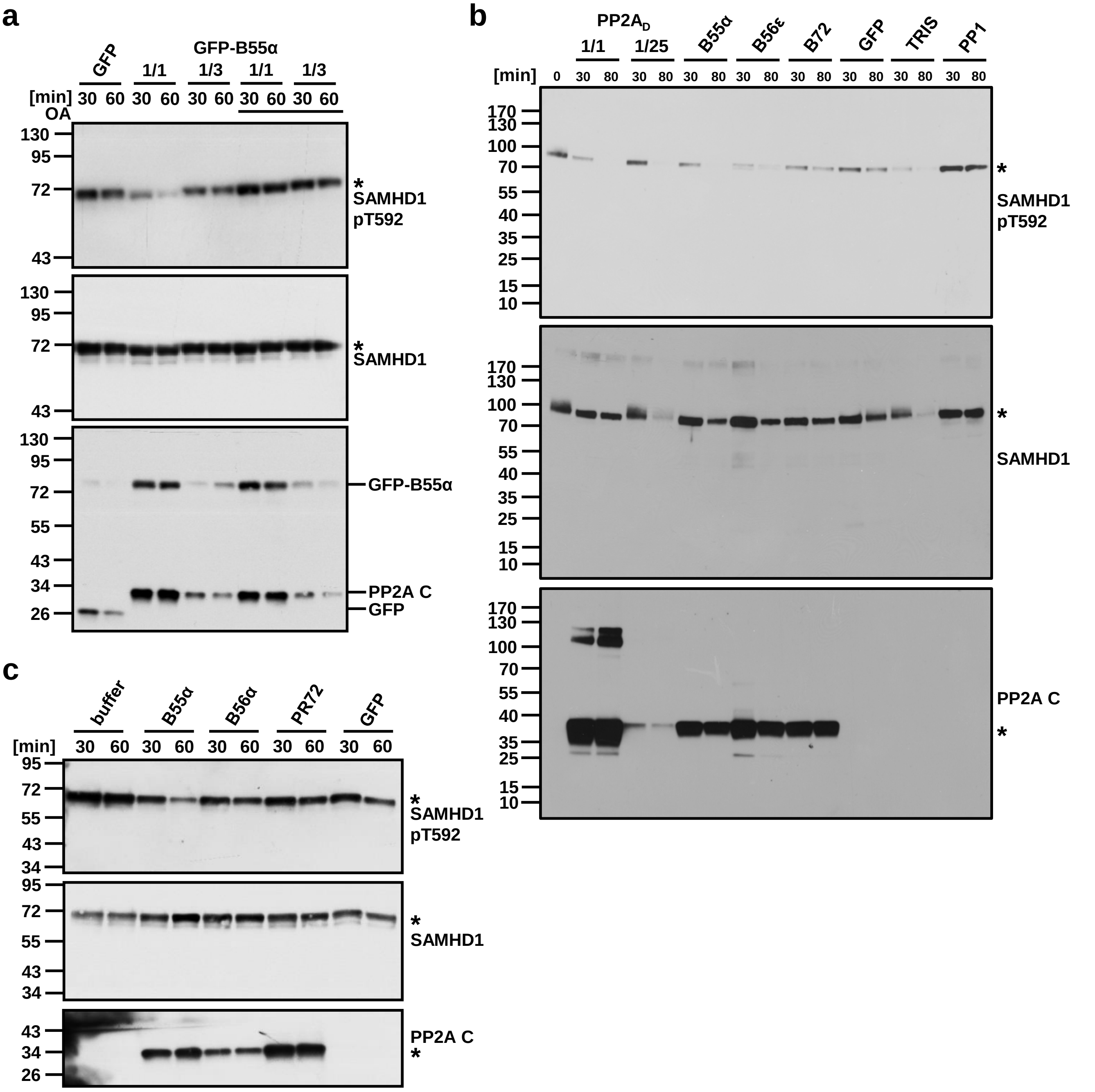

Supplementary Figure 15: Full scans of immunoblots – related to Figure 5a-c.

(a) Full scan for SAMHD1 pT592, SAMHD1, GFP and PP2A C subunit immunoblots shown in Fig. 5a. Signals of respective proteins are marked with asterisks, as membranes were either probed consecutively with different antibodies (in some instances without stripping) or unspecific bands occurred.

(b) Full scan for SAMHD1 pT592, SAMHD1 and PP2A C subunit immunoblots shown in Fig. 5b. Signals of respective proteins are marked with asterisks, as membranes were either probed consecutively with different antibodies (in some instances without stripping) or unspecific bands occurred.

(c) Full scan for SAMHD1 pT592, SAMHD1 and PP2A C subunit immunoblots shown in Fig. 5c. Signals of respective proteins are marked with asterisks, as membranes were either probed consecutively with different antibodies (in some instances without stripping) or unspecific bands occurred. In some instances membranes were cut before probing.

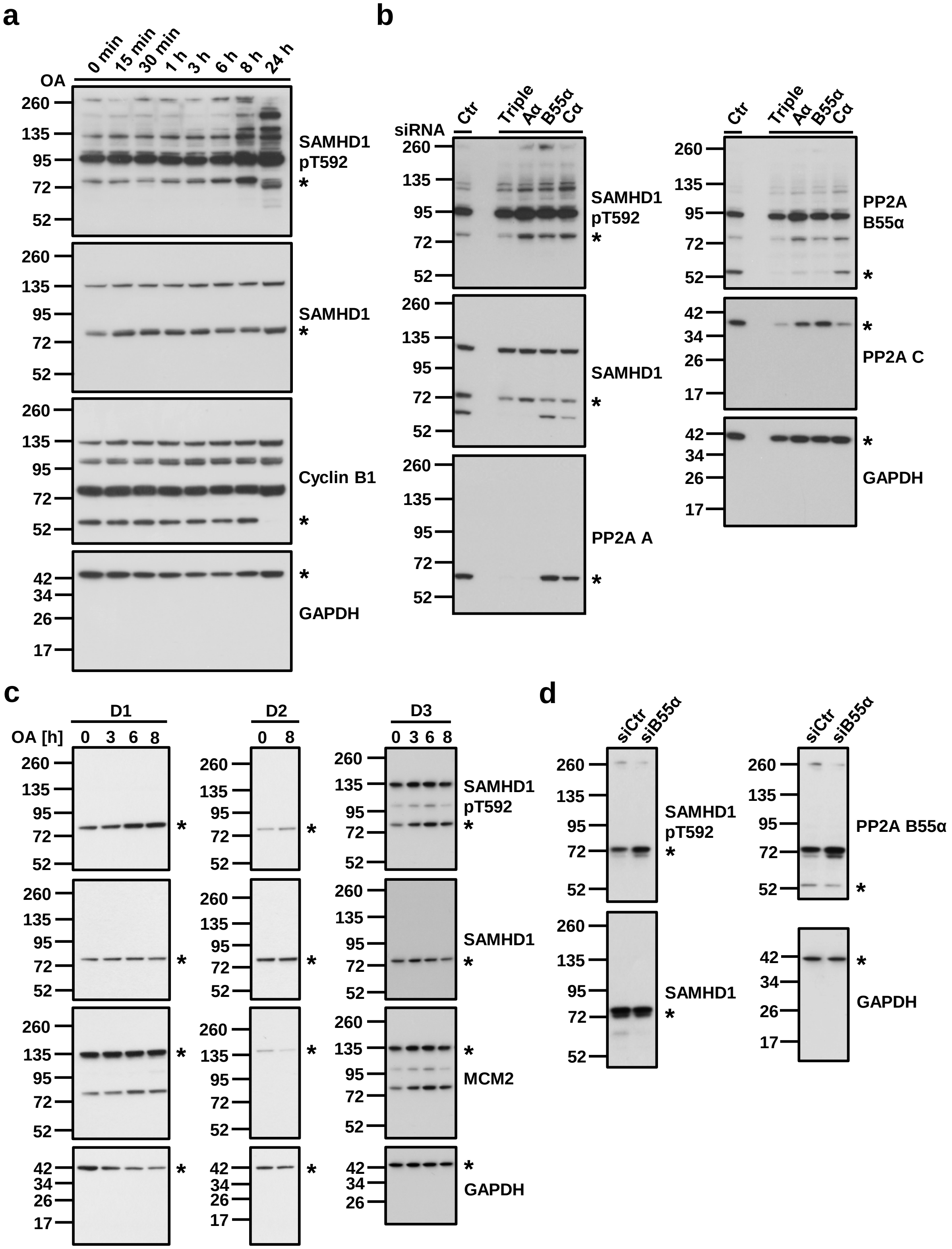
a
OA
0 min
15 min
30 min
1 h
3 h
6 h
8 h
24 h
260
135
95
72
52
42
34
26
17
SAMHD1 pT592
SAMHD1
Cyclin B1
GAPDH
*
b
siRNA
Ctr
Triple
Aα
B55α
Cα
SAMHD1 pT592
SAMHD1
PP2A A
PP2A B55α
PP2A C
GAPDH
c
D1
D2
D3
OA [h]
0
3
6
8
SAMHD1 pT592
SAMHD1
MCM2
GAPDH
d
siCtr
siB55α
SAMHD1 pT592
SAMHD1
PP2A B55α
GAPDH

Supplementary Figure 16: Full scans of immunoblots – related to Figure 5d-g.

(a) Full scan for SAMHD1 pT592, SAMHD1, cyclin B1 and GAPDH immunoblots shown in Fig. 5d. Signals of respective proteins are marked with asterisks, as membranes were either probed consecutively with different antibodies (in some instances without stripping) or unspecific bands occurred. In some instances membranes were cut before probing.

(b) Full scan for SAMHD1 pT592, SAMHD1, PP2A A subunit, PP2A B55α subunit, PP2A C subunit and GAPDH immunoblots shown in Fig. 5e. Signals of respective proteins are marked with asterisks, as membranes were either probed consecutively with different antibodies (in some instances without stripping) or unspecific bands occurred. In some instances membranes were cut before probing.

(c) Full scan for SAMHD1 pT592, SAMHD1, MCM2 and GAPDH immunoblots shown in Fig. 5f. Signals of respective proteins are marked with asterisks, as membranes were either probed consecutively with different antibodies (in some instances without stripping) or unspecific bands occurred. In some instances membranes were cut before probing.

(d) Full scan for SAMHD1 pT592, SAMHD1, PP2A B55α subunit and GAPDH immunoblots shown in Fig. 5g. Signals of respective proteins are marked with asterisks, as membranes were either probed consecutively with different antibodies (in some instances without stripping) or unspecific bands occurred. In some instances membranes were cut before probing.

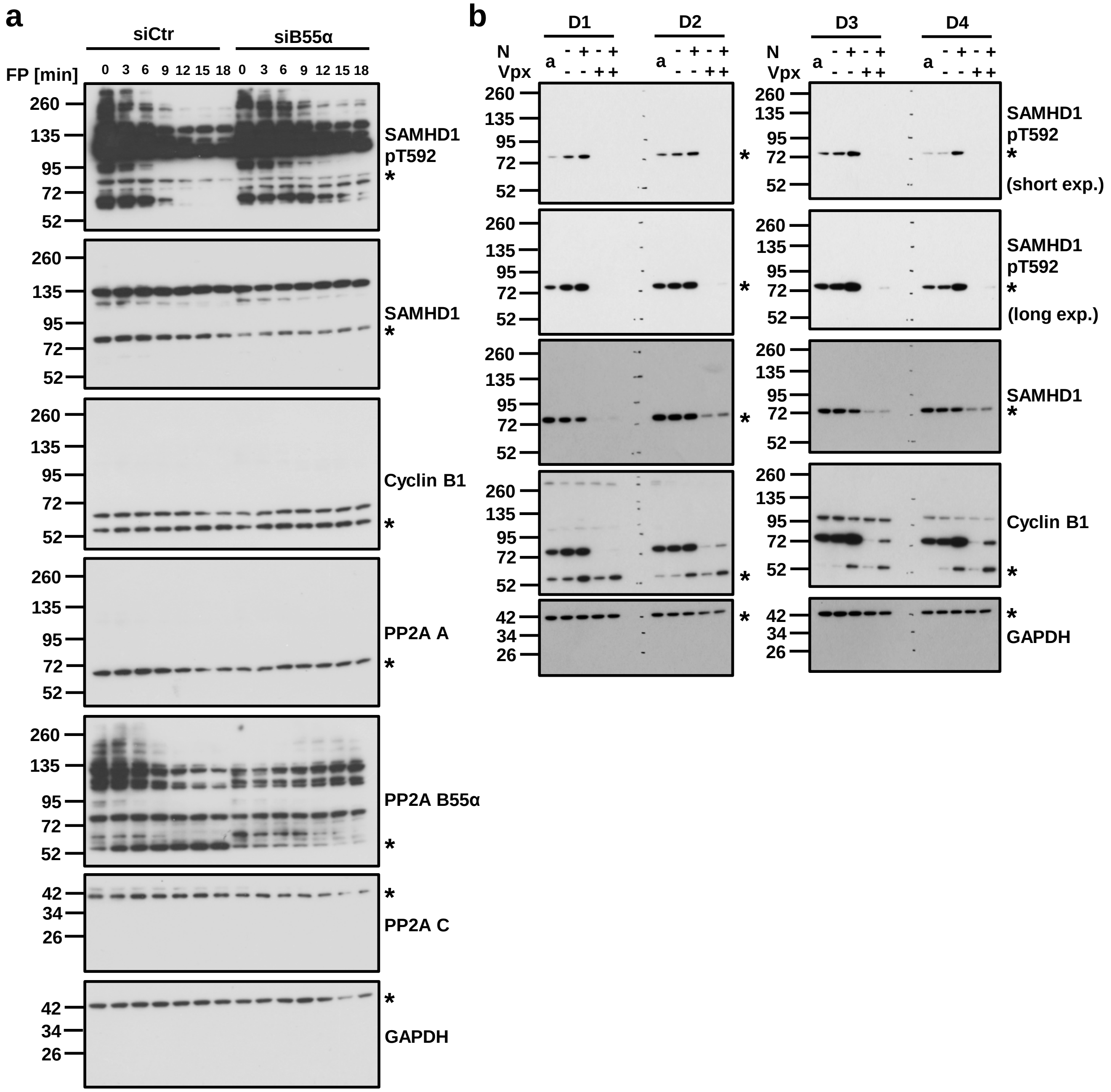

Supplementary Figure 17: Full scans of immunoblots – related to Figure 6 and Figure 7f.

(a) Full scan for SAMHD1 pT592, SAMHD1, cyclin B1, PP2A A subunit, PP2A B55α subunit, PP2A C subunit and GAPDH immunoblots shown in Fig. 6. Signals of respective proteins are marked with asterisks, as membranes were either probed consecutively with different antibodies (in some instances without stripping) or unspecific bands occurred. In some instances membranes were cut before probing.

(b) Full scan for SAMHD1 pT592, SAMHD1, cyclin B1 and GAPDH immunoblots shown in Fig. 7f. Signals of respective proteins are marked with asterisks, as membranes were either probed consecutively with different antibodies (in some instances without stripping) or unspecific bands occurred. In some instances membranes were cut before probing.

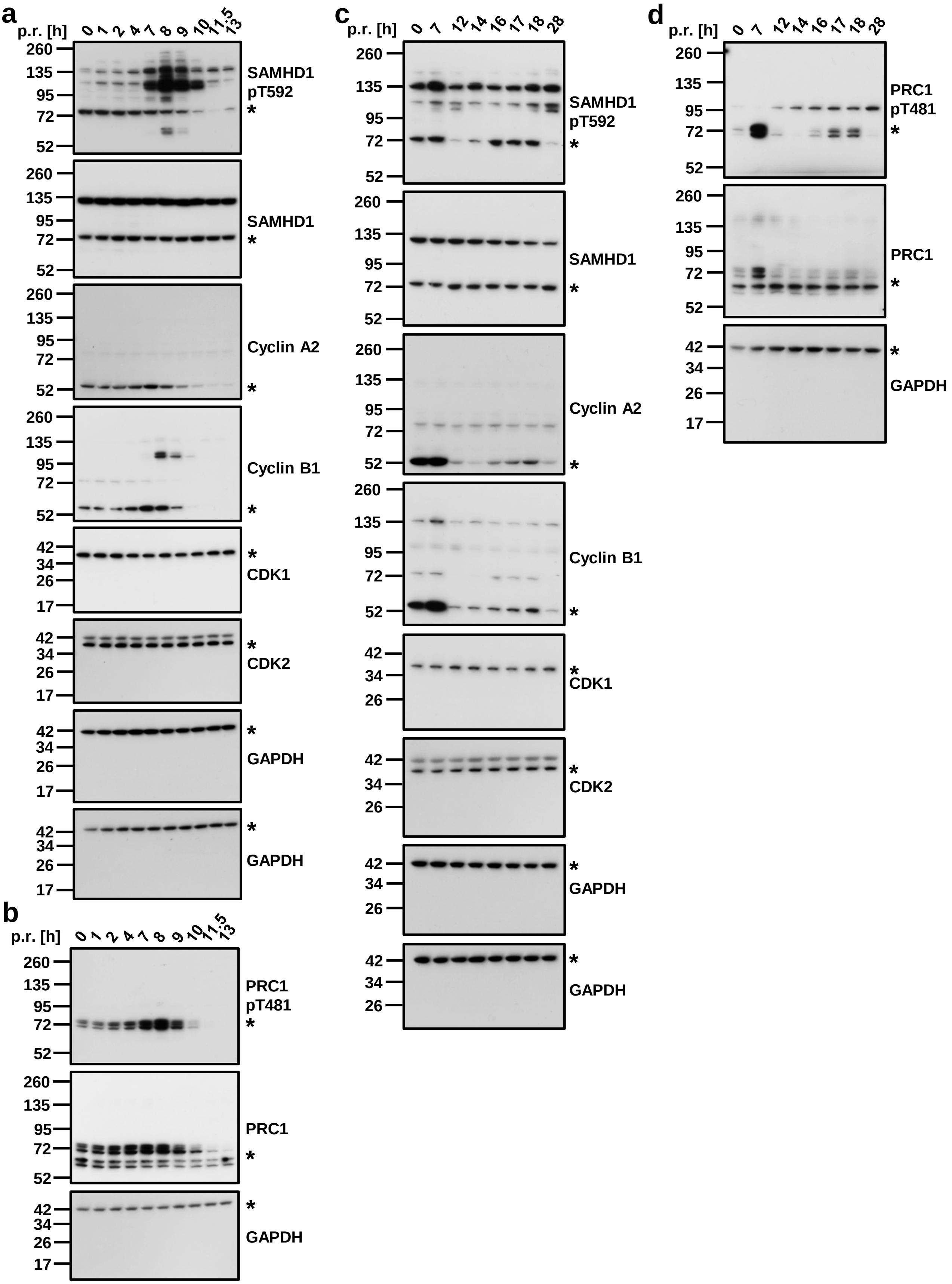
a
p.r. [h]
0 1 2 4 7 8 9 10 11.5 13
260
135
95
72
52
SAMHD1
pT592
*
SAMHD1
Cyclin A2
Cyclin B1
42
34
26
17
CDK1
CDK2
GAPDH
GAPDH
b
p.r. [h]
0 1 2 4 7 8 9 10 11.5 13
PRC1
pT481
PRC1
GAPDH
c
p.r. [h]
0 7 12 14 16 17 18 28
SAMHD1
pT592
SAMHD1
Cyclin A2
Cyclin B1
CDK1
CDK2
GAPDH
GAPDH
d
p.r. [h]
0 7 12 14 16 17 18 28
PRC1
pT481
PRC1
GAPDH

Supplementary Figure 18: Full scans of immunoblots – related to Supplementary Figure 1a, c, d, f.

(a) Full scan for SAMHD1 pT592, SAMHD1, cyclin A2, cyclin B1, CDK1, CDK2 and GAPDH immunoblots shown in Supplementary Fig. 1a. Signals of respective proteins are marked with asterisks, as membranes were either probed consecutively with different antibodies (in some instances without stripping) or unspecific bands occurred. In some instances membranes were cut before probing.

(b) Full scan for PRC1 pT481, PRC1 and GAPDH immunoblots shown in Supplementary Fig. 1c. Signals of respective proteins are marked with asterisks, as membranes were either probed consecutively with different antibodies (in some instances without stripping) or unspecific bands occurred. In some instances membranes were cut before probing.

(c) Full scan for SAMHD1 pT592, SAMHD1, cyclin A2, cyclin B1, CDK1, CDK2 and GAPDH immunoblots shown in Supplementary Fig. 1d. Signals of respective proteins are marked with asterisks, as membranes were either probed consecutively with different antibodies (in some instances without stripping) or unspecific bands occurred. In some instances membranes were cut before probing.

(d) Full scan for PRC1 pT481, PRC1 and GAPDH immunoblots shown in Supplementary Fig. 1f. Signals of respective proteins are marked with asterisks, as membranes were either probed consecutively with different antibodies (in some instances without stripping) or unspecific bands occurred. In some instances membranes were cut before probing.

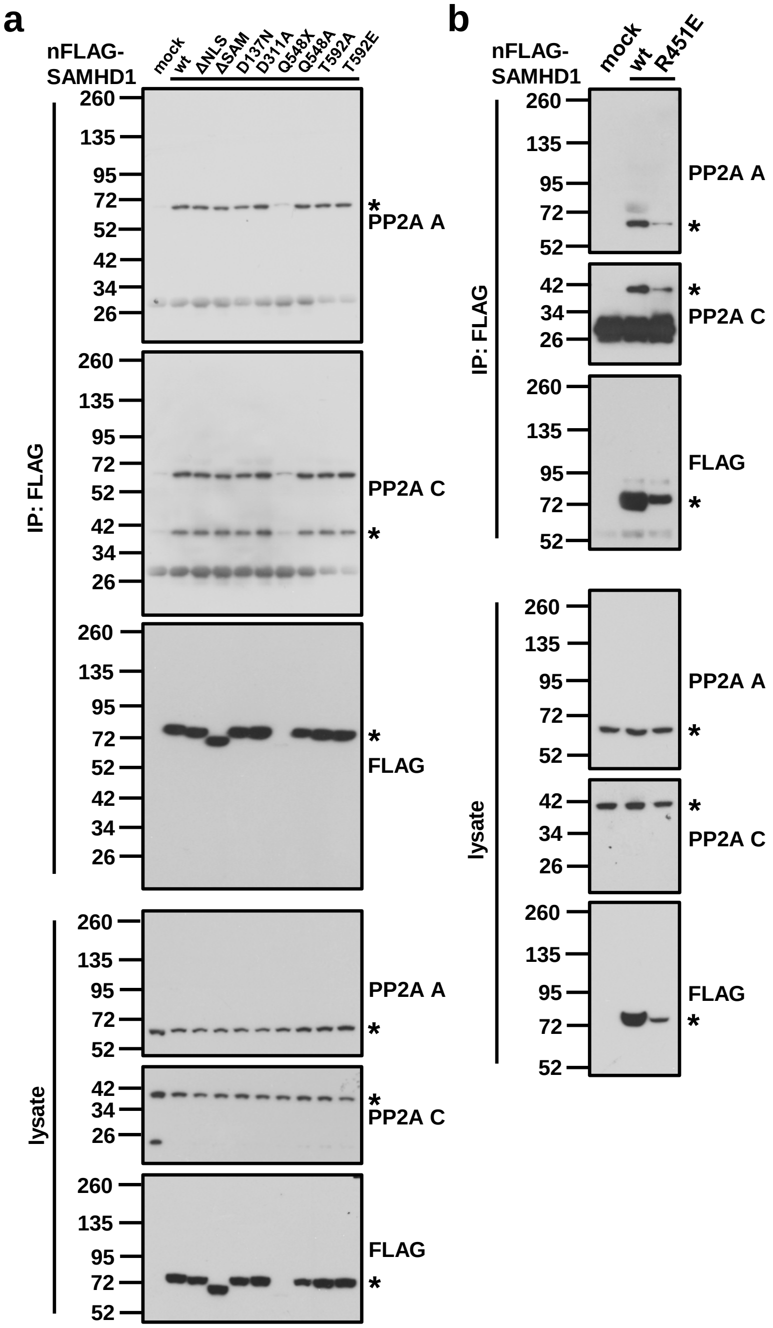

Supplementary Figure 19: Full scans of immunoblots – related to Supplementary Figure 2d, e.

(a) Full scan for PP2A A subunit, PP2A C subunit and FLAG immunoblots (IP + lysate) shown in Supplementary Fig. 2d. Signals of respective proteins are marked with asterisks, as membranes were either probed consecutively with different antibodies (in some instances without stripping) or unspecific bands occurred. In some instances membranes were cut before probing.

(b) Full scan for PP2A A subunit, PP2A C subunit and FLAG immunoblots (IP + lysate) shown in Supplementary Fig. 2e. Signals of respective proteins are marked with asterisks, as membranes were either probed consecutively with different antibodies (in some instances without stripping) or unspecific bands occurred. In some instances membranes were cut before probing.

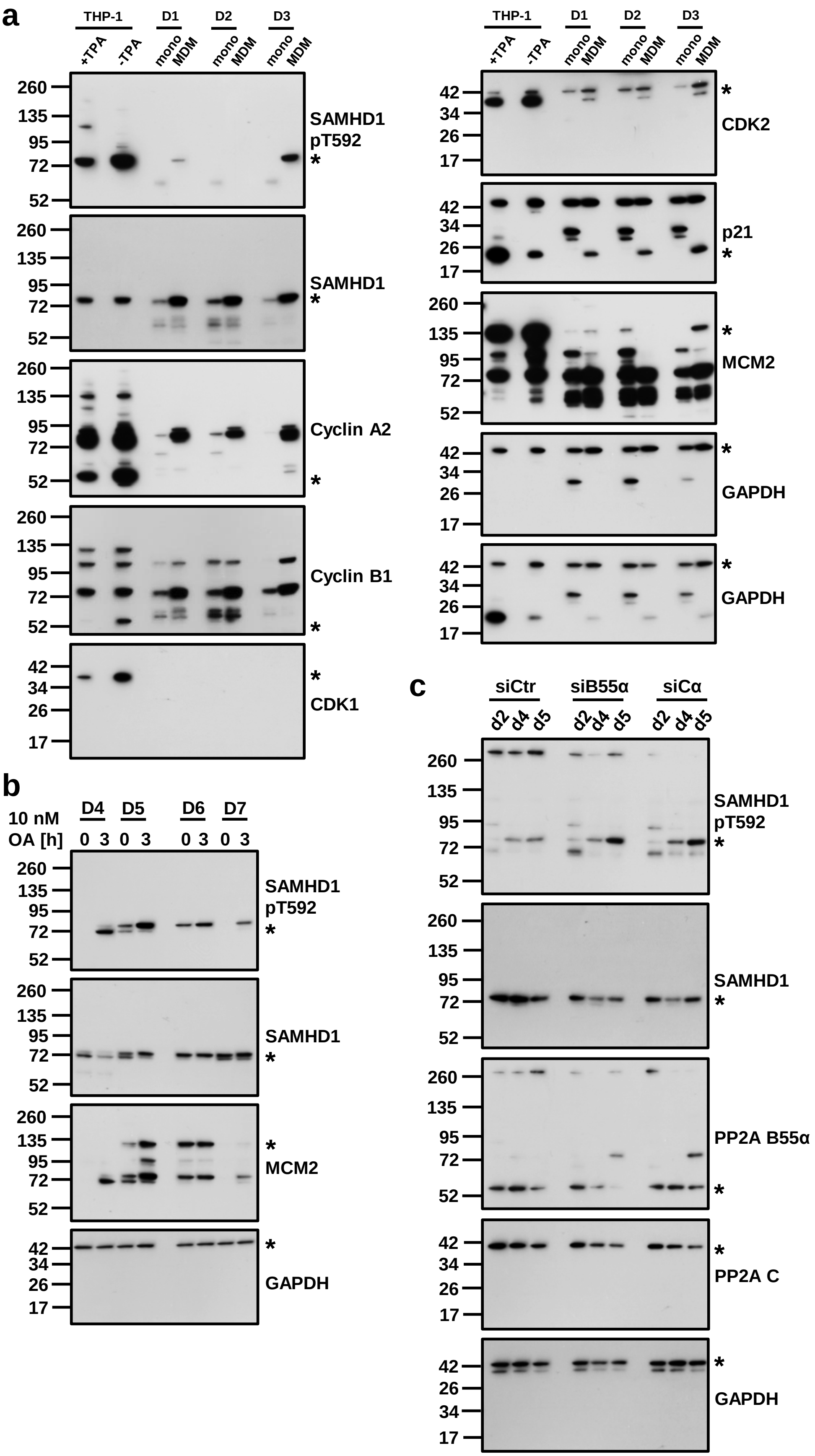
a
THP-1
D1
D2
D3
+TPA
-TPA
mono
MDM
260
135
95
72
52
42
34
26
17
SAMHD1 pT592
SAMHD1
Cyclin A2
Cyclin B1
CDK1
CDK2
p21
MCM2
GAPDH
b
10 nM OA [h]
D4
D5
D6
D7
0
3
c
siCtr
siB55α
siCα
d2
d4
d5
PP2A B55α
PP2A C
*

Supplementary Figure 20: Full scans of immunoblots – related to Supplementary Figure 3b, e, f.

(a) Full scan for SAMHD1 pT592, SAMHD1, cyclin A2, cyclin B1, CDK1, CDK2, p21, MCM2 and GAPDH immunoblots shown in Supplementary Fig. 3b. Signals of respective proteins are marked with asterisks, as membranes were either probed consecutively with different antibodies (in some instances without stripping) or unspecific bands occurred. In some instances membranes were cut before probing.

(b) Full scan for SAMHD1 pT592, SAMHD1, MCM2 and GAPDH immunoblots shown in Supplementary Fig. 3e. Signals of respective proteins are marked with asterisks, as membranes were either probed consecutively with different antibodies (sometimes without stripping) or unspecific bands occurred.

(c) Full scan for SAMHD1 pT592, SAMHD1, PP2A B55α subunit, PP2A C subunit and GAPDH immunoblots shown in Supplementary Fig. 3f. Signals of respective proteins are marked with asterisks, as membranes were either probed consecutively with different antibodies (in some instances without stripping) or unspecific bands occurred. In some instances membranes were cut before probing.

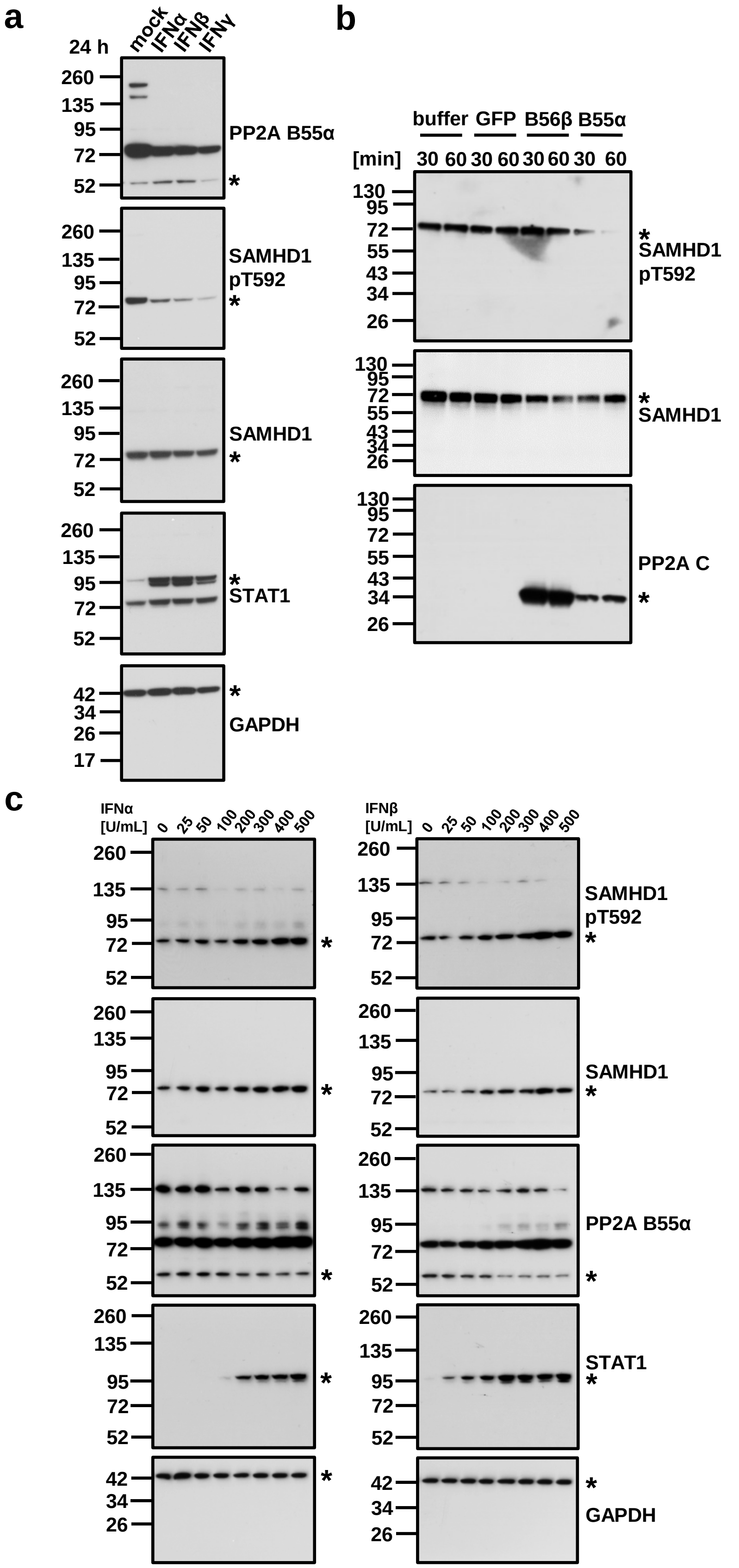
a
24 h
mock
IFNα
IFNβ
IFNγ
260
135
95
72
52
PP2A B55α
*
SAMHD1
pT592
SAMHD1
STAT1
42
34
26
17
GAPDH
b
buffer
GFP
B56β
B55α
[min]
30 60 30 60 30 60 30 60
130
95
72
55
43
34
26
PP2A C
c
IFNα
[U/mL]
0 25 50 100 200 300 400 500
IFNβ
[U/mL]

Supplementary Figure 21: Full scans of immunoblots – related to Supplementary Figure 4b, e, f.
(a) Full scan for PP2A B55α subunit, SAMHD1 pT592, SAMHD1, STAT1 and GAPDH immunoblots shown in Supplementary Fig. 4b. Signals of respective proteins are marked with asterisks, as membranes were either probed consecutively with different antibodies (in some instances without stripping) or unspecific bands occurred. In some instances membranes were cut before probing.
(b) Full scan for SAMHD1 pT592, SAMHD1 and PP2A C subunit immunoblots shown in Supplementary Fig. 4e. Signals of respective proteins are marked with asterisks, as membranes were either probed consecutively with different antibodies (in some instances without stripping) or unspecific bands occurred.
(c) Full scan for SAMHD1 pT592, SAMHD1, PP2A B55α subunit, STAT1 and GAPDH immunoblots shown in Supplementary Fig. 4f. Signals of respective proteins are marked with asterisks, as membranes were either probed consecutively with different antibodies (in some instances without stripping) or unspecific bands occurred. In some instances membranes were cut before probing.

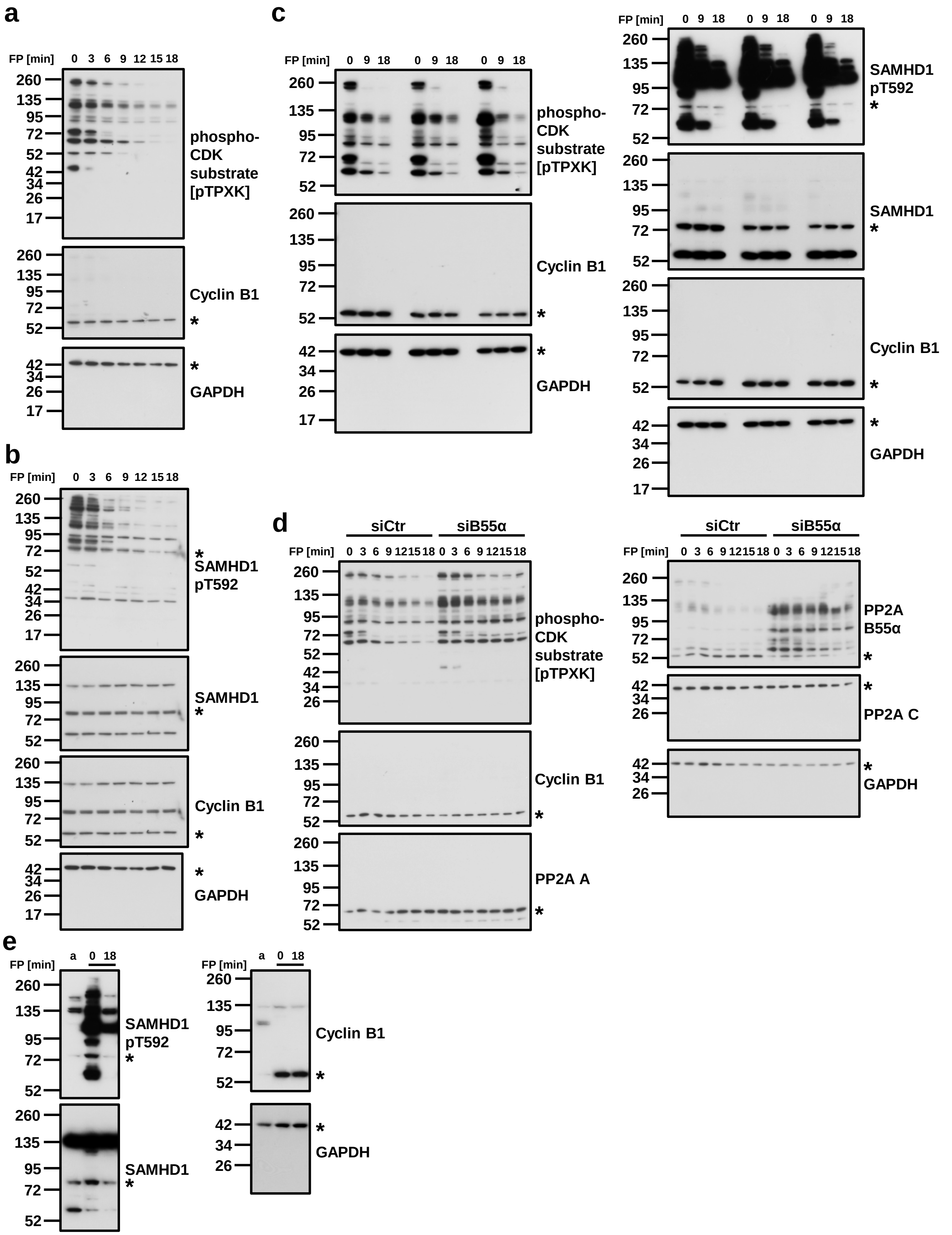
a
FP [min] 0 3 6 9 12 15 18
260 135 95 72 52 42 34 26 17
phospho-CDK substrate [pTPXK]
Cyclin B1
*
GAPDH
b
FP [min] 0 3 6 9 12 15 18
SAMHD1 pT592
SAMHD1
Cyclin B1
GAPDH
*
c
FP [min] 0 9 18 0 9 18 0 9 18
phospho-CDK substrate [pTPXK]
Cyclin B1
GAPDH
SAMHD1 pT592
SAMHD1
d
siCtr
siB55α
FP [min] 0 3 6 9 12 15 18 0 3 6 9 12 15 18
phospho-CDK substrate [pTPXK]
Cyclin B1
PP2A A
PP2A B55α
PP2A C
GAPDH
e
FP [min] a 0 18
SAMHD1 pT592
SAMHD1
Cyclin B1
GAPDH

Supplementary Figure 22: Full scans of immunoblots – related to Supplementary Figure 5a, b, d, e, f.
(a) Full scan for phospho-CDK substrate [pTPXK], cyclin B1 and GAPDH immunoblots shown in Supplementary Fig. 5a. Signals of respective proteins are marked with asterisks, as membranes were either probed consecutively with different antibodies (in some instances without stripping) or unspecific bands occurred. In some instances membranes were cut before probing.
(b) Full scan for SAMHD1 pT592, SAMHD1, cyclin B1 and GAPDH immunoblots shown in Supplementary Fig. 5b. Signals of respective proteins are marked with asterisks, as membranes were either probed consecutively with different antibodies (in some instances without stripping) or unspecific bands occurred. In some instances membranes were cut before probing.
(c) Full scan for phospho-CDK substrate [pTPXK], cyclin B1 and GAPDH immunoblots (left panel) as well as SAMHD1 pT592, SAMHD1, cyclin B1 and GAPDH immunoblots (right panel) shown in Supplementary Fig. 5d. Signals of respective proteins are marked with asterisks, as membranes were either probed consecutively with different antibodies (in some instances without stripping) or unspecific bands occurred. In some instances membranes were cut before probing.
(d) Full scan for phospho-CDK substrate [pTPXK], cyclin B1, PP2A A subunit, PP2A B55α subunit, PP2A C subunit and GAPDH immunoblots shown in Supplementary Fig. 5e. Signals of respective proteins are marked with asterisks, as membranes were either probed consecutively with different antibodies (in some instances without stripping) or unspecific bands occurred. In some instances membranes were cut before probing.
(e) Full scan for SAMHD1 pT592, SAMHD1, cyclin B1 and GAPDH immunoblots shown in Supplementary Fig. 5f. Signals of respective proteins are marked with asterisks, as membranes were either probed consecutively with different antibodies (in some instances without stripping) or unspecific bands occurred. In some instances membranes were cut before probing.

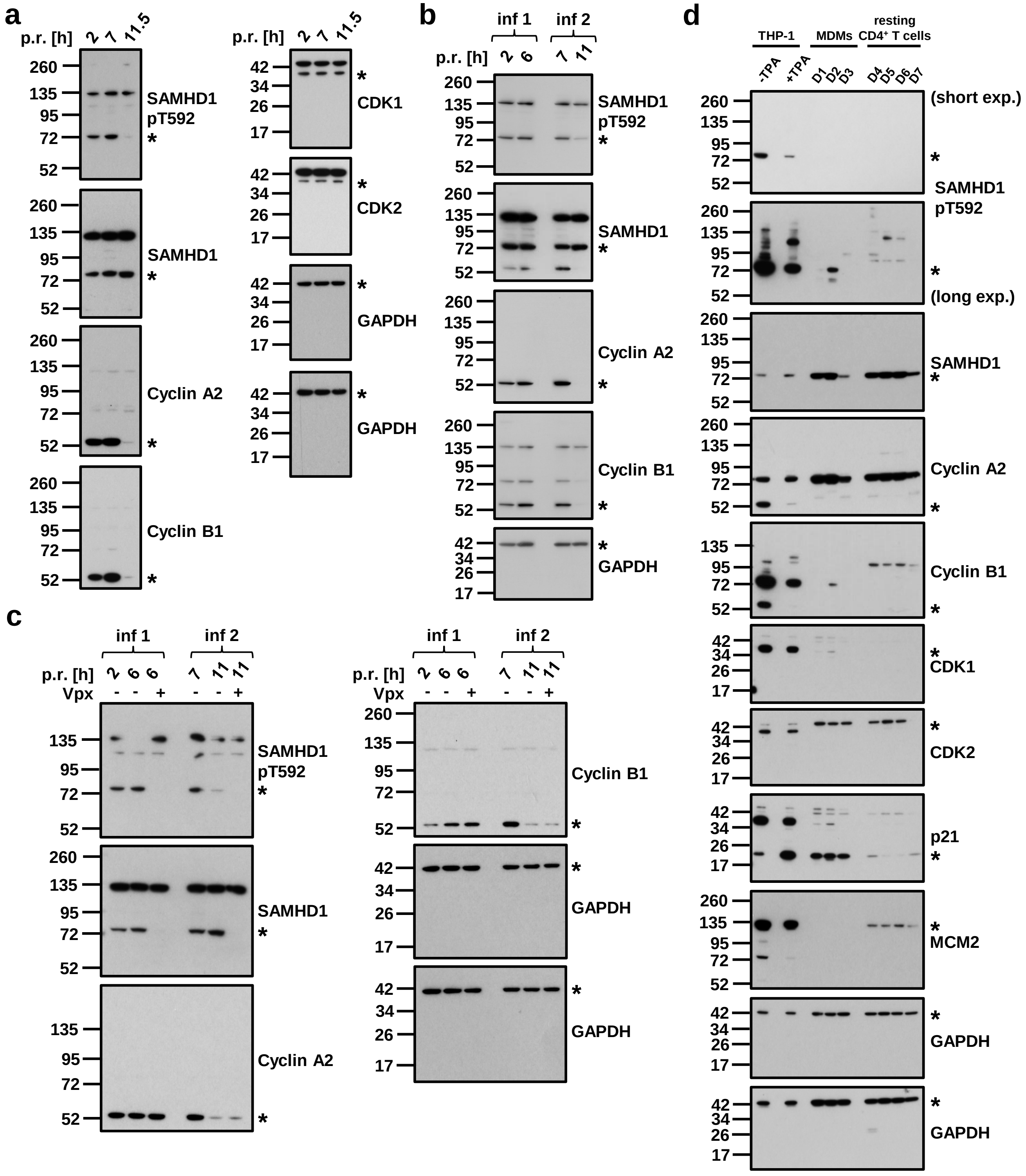
a
p.r. [h]
2
7
11.5
260
135
95
72
52
42
34
26
17
SAMHD1
pT592
SAMHD1
Cyclin A2
Cyclin B1
CDK1
CDK2
GAPDH
*
b
inf 1
inf 2
6
11
c
Vpx
-
+
d
THP-1
MDMs
resting
CD4+ T cells
-TPA
+TPA
D1
D2
D3
D4
D5
D6
D7
(short exp.)
(long exp.)
p21
MCM2

Supplementary Figure 23: Full scans of immunoblots – related to Supplementary Figure 6b, f, h and Figure 7c.

(a) Full scan for SAMHD1 pT592, SAMHD1, cyclin A2, cyclin B1, CDK1, CDK2 and GAPDH immunoblots shown in Fig. 6b. Signals of respective proteins are marked with asterisks, as membranes were either probed consecutively with different antibodies (in some instances without stripping) or unspecific bands occurred. In some instances membranes were cut before probing.

(b) Full scan for SAMHD1 pT592, SAMHD1, cyclin A2, cyclin B1 and GAPDH immunoblots shown in Fig. 6f. Signals of respective proteins are marked with asterisks, as membranes were either probed consecutively with different antibodies (in some instances without stripping) or unspecific bands occurred. In some instances membranes were cut before probing.

(c) Full scan for SAMHD1 pT592, SAMHD1, cyclin A2, cyclin B1 and GAPDH immunoblots shown in Fig. 6h. Signals of respective proteins are marked with asterisks, as membranes were either probed consecutively with different antibodies (in some instances without stripping) or unspecific bands occurred. In some instances membranes were cut before probing.

(d) Full scan for SAMHD1 pT592, SAMHD1, cyclin A2, cyclin B1, CDK1, CDK2, p21, MCM2 and GAPDH immunoblots shown in Supplementary Fig. 7c. Signals of respective proteins are marked with asterisks, as membranes were either probed consecutively with different antibodies (in some instances without stripping) or unspecific bands occurred. In some instances membranes were cut before probing.

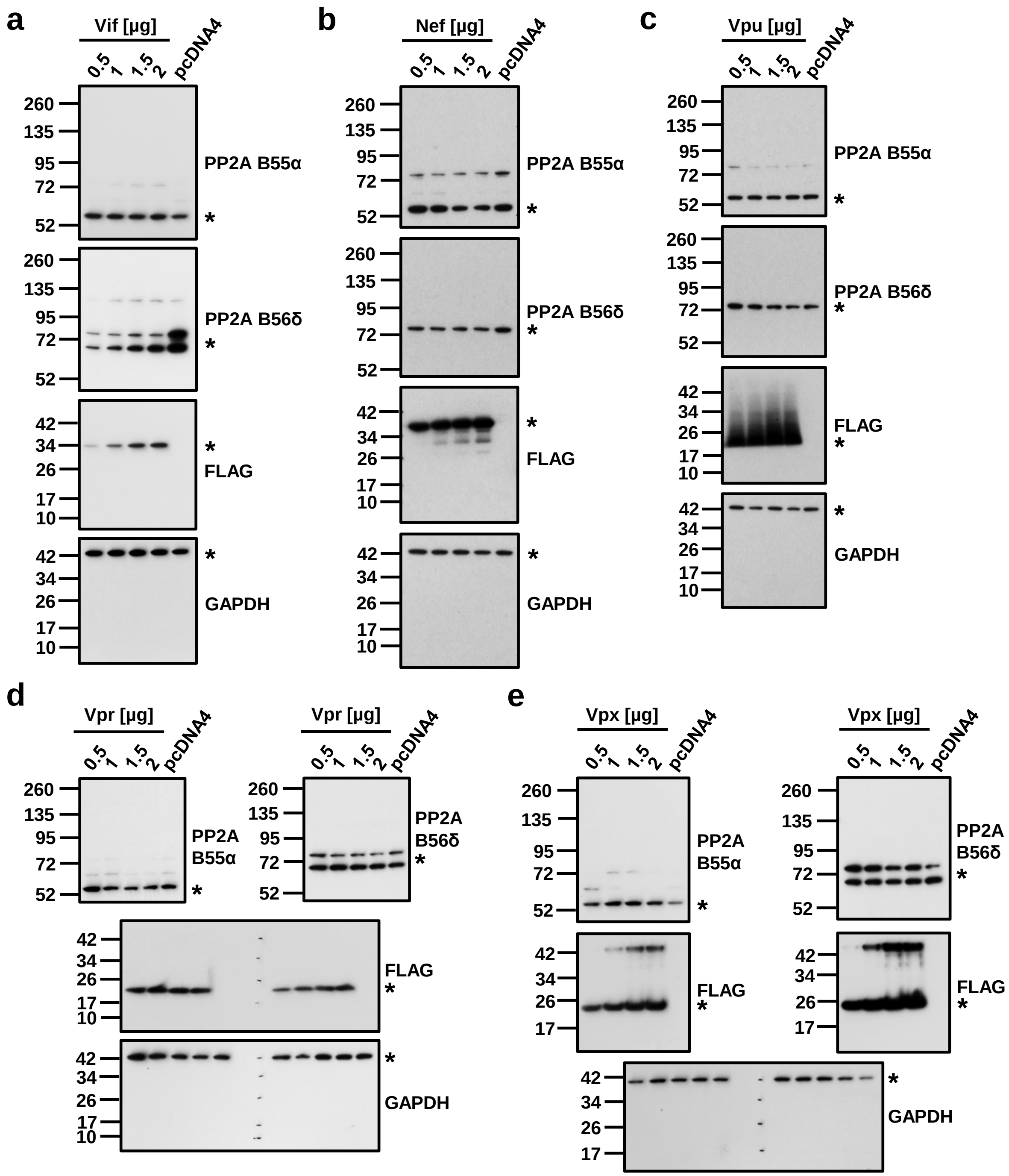

Supplementary Figure 24: Full scans of immunoblots – related to Supplementary Figure 8.
(a)-(e) Full scan for PP2A B55α subunit, PP2A B56δ subunit, FLAG and GAPDH immunoblots shown in Supplementary Fig. 8a-e. Signals of respective proteins are marked with asterisks, as membranes were either probed consecutively with different antibodies (in some instances without stripping) or unspecific bands occurred. In some instances membranes were cut before probing.

Supplementary Table 1: PP2A, not protein phosphatase 1 (PP1), is able to dephosphorylate SAMHD1 pT592 *in vitro* – related to Figure 3.

Targeted MS^2 data concerning SAMHD1 (aa region 577-596) were processed with PinPoint Software 1.4.0 (Thermo Fisher Scientific). Areas under the curve for selective precursor-fragment SRM transitions were calculated. Normalization between different conditions was executed according to a non-phosphorylatable proteotypic peptide of SAMHD1 (GGFEEPVLLK). Calculation of phosphorylation stoichiometry was executed as described in Schreurs *et al*.[41] and based on a method described by Olsen *et al*.[42].

**Peptide**		**Charge**	**m/z Precursor**	**m/z Fragment**	**Ion type**	**Area**			**Area (after normalization to control)**			**Ratio**		
						**Control**	**PP2A**	**PP1**	**Control**	**PP2A**	**PP1**	**Control/ control**	**PP2A/ control**	**PP1/ control**
GGFEEPVLLK	aa 57-66	2+	544.80	569.40	y5	1.89×10^{8}	1.71×10^{8}	2.02×10^{8}	1.89×10^{8}	1.89×10^{8}	1.89×10^{8}	1.0	1.0	1.0
NFTKPQDGDVIAPLITPQK	aa 577-595	3+	694.71	796.49	y7	1.64×10^{7}	8.34×10^{7}	3.19×10^{7}	1.64×10^{7}	9.23×10^{7}	2.98×10^{7}	1.0	5.6	1.8
NFTKPQDGDVIAPLI**T[p]**PQK		3+	721.37	876.46	y7 (+phos)	3.88×10^{6}	2.33×10^{5}	6.82×10^{6}	3.88×10^{6}	2.58×10^{5}	6.36×10^{6}	1.0	0.1	1.6
NFTKPQDGDVIAPLITPQKK	aa 577-596	4+	553.31	924.59	y8	1.17×10^{7}	3.20×10^{7}	1.24×10^{7}	1.17×10^{7}	3.54×10^{7}	1.16×10^{7}	1.0	3.0	1.0
NFTKPQDGDVIAPLI**T[p]**PQKK		3+	764.07	1004.55	y8 (+phos)	1.30×10^{8}	7.29×10^{6}	1.53×10^{8}	1.30×10^{8}	8.06×10^{6}	1.43×10^{8}	1.0	0.1	1.1
PQDGDVIAPLITPQK	aa 581-595	2+	796.44	796.49	y7	2.80×10^{5}	8.01×10^{5}	3.61×10^{5}	2.80×10^{5}	8.86×10^{5}	3.37×10^{5}	1.0	3.2	1.2
PQDGDVIAPLI**T[p]**PQK		2+	836.42	876.46	y7 (+phos)	1.17×10^{5}	0	1.16×10^{5}	1.17×10^{5}	0	1.08×10^{5}	1.0	0.0	0.9

Supplementary Note 1

The discovery of PP2A-B55α dephosphorylating SAMHD1 at T592 is in line with current knowledge on the timely coordinated and conserved program that occurs during mitotic exit in mammalian cells[1]. During mitotic entry, CDK1/ cyclin B activates the kinase Greatwall-MASTL, which in turn phosphorylates the PP2A-B55 inhibitors, small proteins of the Endosulfine family (Endos), endosulfine α (ENSA) and ARPP19[2,3]. As long as PP2A-B55α is kept inactive by Greatwall-phosphorylated Endos, SAMHD1 is phosphorylated until mitotic exit events start to unfold. PP2A-B55 inhibition is maintained until the spindle assembly checkpoint is satisfied and the ubiquitin E3 ligase APC/C triggers degradation of cyclin B and securin, which is accompanied by rephosphorylation of inhibitory residues on CDK1. The resulting drop in CDK1 activity leads to the reactivation of CDK1-counteracting phosphatases. These events result in the ordered removal of mitotic phosphorylations initiating mitotic exit. PP1[4–7], but also the Fcp phosphatase and PP2A-B55 itself[8–10], all contribute to inactivate Greatwall-MASTL which in turn leads to reduction of phosphorylation on Endos, relieving the inhibition on PP2A-B55. Phosphorylated Endos acts both as an inhibitor and a substrate of PP2A-B55, and competes with other CDK-phosphorylated mitotic substrates for access to PP2A-B55. Both, the difference in kinetic parameters of PP2A-B55 for phosphorylated Endos over other substrates, and additionally the molar excess of Endos over PP2A-B55 can explain this conundrum[8,11]. The mechanism called “inhibition by unfair competition” explains also how phosphorylated Endos itself becomes inactivated[8,11]. As soon as Greatwall is inactivated, phosphorylated Endos will not be replaced and the inhibition of PP2A-B55 is removed. This will result in a constitutive B55 activity during mitotic exit, leading to dephosphorylation of CDK1 substrates (including SAMHD1) and finally to spindle disassembly, nuclear envelope reformation and cytokinesis. With the beginning of S phase, active CDK2/ cyclin A2 complexes phosphorylate SAMHD1 at T592, further maintained by activated CDK1/ cyclin A2 complexes until mitosis, reflecting our results that demonstrate a constant phosphorylation signal during S/ G_2/ M phase (Fig. 1 and Supplementary Fig. 1)[12–14]. In summary, consistent with our observations, the key mitotic exit phosphatase in mammalian cells, PP2A-B55α[15], is reactivated at the end of mitosis by inhibition of the ENSA/ Greatwall pathway resulting in dephosphorylated SAMHD1 at T592 only in the G_1 phase of proliferating cells.

The key residues we determined relevant for binding of human SAMHD1 to PP2A (residues 559, 566, 596, 609, 611, 622) are situated in the C-terminus of SAMHD1. The C-terminus has been shown to serve as a platform for interaction with cellular and viral partners[16–18]. The structural model indicates that the C-terminus must be highly accessible in monomeric as well as tetrameric formation (see Supplementary Fig. 9 and [19]). The C-terminus is comprised of α-helices (observed density up to residue 599) separated from the core HD domain by a three-folded β-sheet. Additional residues 606-624 were resolved in a ternary complex including Vpx_{sm}, DCAF1 and the C-terminus of SAMHD1 forming two α-helices connected by a loop-structure[20]. We indicated the residues that we determined relevant for binding with PP2A (see Supplementary Fig. 9) and conclude that they would be easily accessible for PP2A-B55α binding. Binding of Vpx might not affect binding to PP2A as (i) the only two identified shared residues (609 and 622) for binding to both, Vpx or PP2A, were not defined as "key residues" for Vpx binding by Ahn *et al.*[16,20] and (ii) more importantly, subcellular localization of both proteins may prevent interference by binding to nuclear SAMHD1 since B55α is primarily localized to the cytoplasm[21,22]. We speculate that during mitotic exit after nuclear envelope breakdown, PP2A-B55α holoenzymes and SAMHD1 might get in contact and allow for rapid SAMHD1 dephosphorylation at T592 as observed in our study (Supplementary Fig. 5b). However, the spatial and temporal context of regulation is not clear for non-cycling cells, as macrophages and resting $CD4^+$ T cells seem to harbor a significant portion of SAMHD1 in both the cytoplasm and nucleus[23,24].

Intriguingly, PP2A B55α subunit was specifically upregulated by IFNs in MDMs. Indeed, this is the first study reporting the IFN-inducibility of PP2A subunits. So far, exogenous stimuli have been reported to affect PP2A by directly regulating its activity, mostly through post-translational modifications of the holoenzyme (reviewed in Lambrecht *et al.*[22]). At this point, we cannot exclude that other PP2A holoenzymes might act on SAMHD1 after IFN treatment as well. Additionally, post-translational modifications of PP2A might occur that alter its phosphatase activity to different extents, depending on the type of IFN applied. Moreover, IFN might lead to upregulation of PP2A-regulating proteins that enhance the activity of PP2A on SAMHD1 (e.g. Long *et al.*[25] and Okamoto *et al.*[26]). Therefore, future studies would be needed to characterize PP2A activity after IFN stimulation in more detail. Specific phosphatase activation through IFNs would allow for an additional mechanism to control SAMHD1 function in different

immunological and cellular contexts. Moreover, the upregulation of phosphatase subunits by IFNs may indicate an extra layer of the antiviral IFN response, consequently altering the activity of antiviral factors.

Supplementary Methods

MDM characterization by flow cytometry

2 x 10^5 MDMs were collected by centrifugation (all centrifugation steps were performed at 254 g, 4 °C, 7 min). The supernatant was aspirated and cells were washed twice in FACS staining-buffer (PBS containing 10 % FBS) before incubation with specific antibodies for 1 h, on ice, in a final volume of 50 µL. Antibodies against cellular surface proteins CD14, CD163, CD206 and corresponding isotype controls are listed in the table below. After incubation with antibodies, MDMs were washed twice with FACS staining-buffer and fixed in 2 % paraformaldehyde (PFA) at RT for 30 min. PFA was removed by two washing steps and MDMs were analyzed using the BD LSR II flow cytometer (BD Biosciences).

Antibody	Company	Clone	Volume per sample [µL]	Fluorochrome
CD14	BioLegend	M5E2	1	Pacific Blue
CD163	BD Biosciences	GHI/61	2.5	PE
CD206	BD Biosciences	19.2	2	APC
IgG2a, κ isotype control	BioLegend	MOPC-173	1	Pacific Blue
IgG1 isotype control	Beckman Coulter	679.1Mc7	2	PE
IgG1, κ isotype control	BD Biosciences	MOPC-21	2	APC

Okadaic acid (OA) treatment of HeLa 'Kyoto' cells/ MDMs

To specifically inhibit PP2A, the phosphatase inhibitor okadaic acid (OA) was used (2-20 nM). For this, 4 x10^5 HeLa 'Kyoto' cells were seeded in 2 mL DMEM/ 6-well and treated with 2 nM OA (CST) up to 8 h at 37 °C (for immunoblot analysis). Accordingly, 3 x 10^5 MDMs were seeded in 500 µL RPMI-1640 (supplemented with 10 % FBS, 2 mM L-glutamine, 10 mM HEPES, 1 mM sodium pyruvate)/ 24-well and treated with 2 nM OA up to 8 h (for immunoblot analysis) at 37 °C.

RT-qPCRs

Total RNA from MDMs was isolated using the RNeasy Plus Mini Kit (QIAGEN) or NucleoSpin RNA Kit (Macherey-Nagel). Expression of mRNAs was determined in a 384-well format using QuantiTect SYBR Green RT-PCR Kit (QIAGEN) on an ABI7900 cycler (Applied Biosystems). The PCR conditions were as follows: one cycle of reverse transcription for 30 min at 50 °C, initial denaturation/ heat activation for 15

min at 95 °C, followed by 40 cycles of denaturation for 15 sec at 94 °C, annealing for 1 min at 56 °C, elongation for 45 sec at 72 °C. Dissociation analysis was performed subsequently for 15 sec at 95 °C, followed by 15 sec at 60 °C and 15 sec at 95 °C. Used primers are listed in the table below. Primer efficiencies were tested with ten-fold dilution series of the respective RNA template and were >90 %. Each sample was measured in technical triplicates. Data were normalized to mRNA levels of the reference gene *RPL13A*.

qPCR primer pairs were designed to specifically amplify the respective B-type subunits/ isoforms of PP2A. Each primer pair was tested against expression plasmids of GFP-tagged PP2A B-type subunits (0.6 ng plasmid DNA/ reaction) of all other subunits using the iTaq™ Universal SYBR Green Supermix (BioRad). PCR products were analyzed on 2 % (w/v) agarose gels.

Name	Sequence (5' → 3')	Tm [°C]	Length [bp]	Gene description	Reference
hISG54 fwd	CAGCTGAGAATTGCACTGCAA	58	21	*ISG54 (IFIT2)*	[27]
hISG54 rev	GTAGGCTGCTCTCCAAGGAA	60	20		
hPPP2R2A fwd	CTACGAGTGCCAGTCTTTAG	59	20	*B55α (PPP2R2A)*	NM_002717.3
hPPP2R2A rev	GATGCCCTCATGTCACATAG	59	20		
hPPP2R5A fwd	GAACGTGACTTCCTGAAGAC	60	20	*B56α (PPP2R5A)*	NM_001199756.1
hPPP2R5A rev	CTGATCACTGGCTCTGTTAG	60	20		
hPPP2R5B fwd	CCTCTCAGTTCCGCTATCAG	61	20	*B56β (PPP2R5B)*	NM_006244.3
hPPP2R5B rev	GCTCAAGATTGGGCTCATCC	62	20		
hPPP2R5C fwd	TCAGTGTTGCGTCCTCTTTG	62	20	*B56γ (PPP2R5C)*	NC_000014.9
hPPP2R5C rev	CAGGCTGCTTCTAACGTTGG	63	20		
hPPP2R5D fwd	TCATGTTCCCTGCACTCTAC	61	20	*B56δ (PPP2R5D)*	NM_001270476.1
hPPP2R5D rev	CTCTTCAGAAGCTGGATGTC	59	20		
hPPP2R5E fwd	CACTGTCAGGAGCTTATCAC	59	20	*B56ε (PPP2R5E)*	NR_104104.1
hPPP2R5E rev	GATGGGAAGGATGACGTTAG	59	20		
hRPL13A fwd	CCTGGAGGAGAAGAGGAAAGAGA	55	23	*RPL13A*	[27]
hRPL13A rev	TTGAGGACCTCTGTGTATTTGTCAA	55	25		

Plasmids

The generation of a pcDNA3.1(+)-based plasmid for expression of codon-optimized, N-terminally FLAG-tagged SAMHD1 was described previously[28]. Mutations were introduced into codon-optimized SAMHD1

(plasmid template: pcDNA3.1(+)-nFLAG-SAMHD1) via PCR using specific primers containing the respective nucleotide changes, see primer list below. For PCRs, *Pfu*Ultra DNA Polymerase (Agilent Technologies) or KOD Hot Start DNA Polymerase (Merck Millipore) were used according to the manufacturer's instructions. PCR products were treated with *Dpn*I (Promega or NEB) for 1 h at 37 °C, in order to remove non-mutated/ parental template DNA. After transformation into chemically competent *E. coli* cells, single clones were picked and plasmid DNA was isolated (GeneJET Plasmid Miniprep Kit, Thermo Fisher). Introduction of mutations was verified by sequencing. In order to exclude mutations in the plasmid backbone after PCR amplification, the SAMHD1 mutant-encoding inserts were cloned back into pcDNA3.1(+) using *Bam*HI and *Eco*RI (both NEB).

Deletion of the SAM domain (aa 46-110) in SAMDH1 was achieved via fusion PCR using specific, overlapping primers. In the first PCR reactions, the SAMHD1 sequences flanking the SAM domain were amplified (= PCR1 with P1 + P2; PCR2 with P3 + P4) using the *Pfu*Ultra DNA Polymerase according to the manufacturer's instructions. In the following PCR reaction, 50 ng of the PCR products each were used as templates (= PCR3 with P1 + P4). The fused PCR fragment was cloned into pcDNA3.1(+) using *Bam*HI and *Eco*RI. Deletion of the SAM domain was confirmed by restriction analysis and sequencing.

Introduced mutation	Sequence (5' → 3')	Orientation	Plasmid name
ΔNLS	gagagccgatagcgaggacagccccagaaccc	forward	pcDNA3.1(+)-nFLAG-SAMHD1 ΔNLS
	gggttctggggctgtcctcgctatcggctctc	reverse	
ΔSAM	gtacgggccagatatacgcg	forward (P1)	pcDNA3.1(+)-nFLAG-SAMHD1 ΔSAM
	tggtgtccacgccccaggtcttgtagtcggg	reverse (P2)	
	gacctggggcgtggacaccatgaaggtcatcaac	forward (P3)	
	gaaaggcgtcggtgatcatgg	reverse (P4)	
D137N	gtccggatcatcaacaccccccagttc	forward	pcDNA3.1(+)-nFLAG-SAMHD1 D137N
	gaactggggggtgttgatgatccggac	reverse	
D311A	cggcatcgacgtggccaagtgggactact	forward	pcDNA3.1(+)-nFLAG-SAMHD1 D311A
	agtagtcccacttggccacgtcgatgccg	reverse	
R451E	cagatcgagtacgagaacctgtttaag	forward	pcDNA3.1(+)-nFLAG-SAMHD1 R451E
	cttaaacaggttctcgtactcgatctg	reverse	
Q548A	agttcgccgaggcgctgatccggg	forward	pcDNA3.1(+)-nFLAG-SAMHD1 Q548A
	cccggatcagcgcctcggcgaact	reverse	

Q548X	agaagttcgccgagtagctgatccgggtg	forward	pcDNA3.1(+)-nFLAG-SAMHD1 Q548X
	cacccggatcagctactcggcgaacttct	reverse	
T592A	gtgatcgcccctctgatcgcccccagaaaaaagag	forward	pcDNA3.1(+)-nFLAG-SAMHD1 T592A
	ctcttttttctgggggcgatcagaggggcgatcac	reverse	
T592E	gtgatcgcccctctgatcgaaccccagaaaaaagag	forward	pcDNA3.1(+)-nFLAG-SAMHD1 T592E
	ctcttttttctggggttcgatcagaggggcgatcac	reverse	

To identify basic residues in SAMHD1 important for PP2A-B55α binding, the C-terminal region of human (Hs) and murine (isoform 1; Mm) SAMHD1 were compared. Alignment of SAMHD1 protein sequences was generated with ClustalW[29].

In order to change basic residues in the C-terminal region of SAMHD1, mutations were sequentially introduced into SAMHD1 (plasmid template: pcDNA3.1(+)-nFLAG-SAMHD1) as described above. The specific primers containing the respective nucleotide changes and name of the resulting plasmids are listed below.

Introduced mutation	Sequence (5' → 3')	Orientation	Plasmid name
R559G	caagaaggtggacggcaagtccctgtacgccgc	forward	pcDNA3.1(+)-nFLAG-SAMHD1 Hs·Mm
	gcggcgtacagggacttgccgtccaccttcttg	reverse	
R566G	gccgccggccagtacttcgtgc	forward	
	gaagtactggccggcggcgtacagg	reverse	
K596 insertion	cacccccagaaagagtggaacgacagcac	forward	
	gtgctgtcgttccactctttctgggggtg	reverse	
R609C_R611Q	gcaccagcgtgcagaaccccacctgcctgcaggaggccagc	forward	
	gctggcctcctgcaggcaggtggggttctgcacgctggtgc	reverse	
K622F	gtgcagctgttcttcgacgaccccatgtgag	forward	
	ctcacatggggtcgtcgaagaacagctgcac	reverse	

Additionally, mutations were sequentially introduced into murine SAMHD1 (plasmid template: pcDNA3.1(+)-nFLAG-mSAMHD1; protein sequence Acc. No. Q60710-1) as described above. The specific primers containing the respective nucleotide changes and name of the resulting plasmid are listed below.

Introduced mutation	Sequence (5’ → 3‘)	Orientation	Plasmid name
G570R	taagaagaaagaccggaagagcctggacgccg	forward	pcDNA3.1(+)-nFLAG-mSAMHD1 Mm·Hs
	cggcgtccaggctcttccggtctttcttctta	reverse	
G577R	gagcctggacgccgcccggaagcactttgttc	forward	
	gaacaaagtgcttccgggcggcgtccaggctc	reverse	
K607 insertion	cataacacctctgaaaaagtggaataataagac	forward	
	gtcttattattccactttttcagaggtgttatg	reverse	
C614R_Q616R	gaataataagacttcatctcggctccgggaagtatccaaag	forward	
	ctttggatacttcccggagccgagatgaagtcttattattc	reverse	
F627K	catgtctaaaaaagtaaggaattctgcagatatc	forward	
	gatatctgcagaattccttactttttagacatg	reverse	

To generate N-terminally CBP-SBP-tagged SAMHD1, the sequence of codon-optimized SAMHD1 was amplified using specific primers to introduce restriction sites (see the primer listed below). The PCR product was digested and cloned into the *Bam*HI/ *Eco*RI restriction sites of pNTAP-B (Agilent Technologies).

To generate N-terminally GFP-tagged SAMHD1, the sequence of codon-optimized SAMHD1 was amplified using specific primers to introduce restriction sites (see the primer listed below). The PCR product was digested and cloned into the *Eco*RI/ *Bam*HI restriction sites of pEGFP-C1 (Clontech).

Introduced restriction site	Sequence (5’ → 3‘)	Orientation	Plasmid name
5’-*Bam*HI	gcgcggatcccagagagccgatagcgagcag	forward	pNTAPB-SAMHD1
3’-*Eco*RI	cggccgaattctcacatggggtcgtc	reverse	
5'-*Eco*RI	cggccgaattcccagagagccgatagcgagcag	forward	pEGFP-C1-SAMHD1
3'-*Bam*HI	gcgcggatcctcacatggggtcgtccttgaac	reverse	

Eukaryotic expression vectors for N-terminally GST-tagged PP2A subunits B55α, B56α, B56β, B56γ, B56δ, B56ε and PR72, all in pGMEX-T1 (GE Healthcare), have previously been described[30]. Human B55α, B56α and B56β cDNAs were PCR-amplified using Pwo DNA polymerase (Roche), digested with *Bam*HI/ *Eco*RI, *Bam*HI and *Bgl*II respectively, and subsequently cloned into pEGFP-C1 vector (Clontech) digested with *Bgl*II/ EcoRI, *Bam*HI and *Bam*HI respectively. The same strategy was used for cloning of

B55α (*Bam*HI/ *Eco*RI) in pEGFP-N1 (Clontech) (*Bgl*II/ *Eco*RI). The pEGFP-C1 plasmid expressing GFP-tagged PR72 subunit was described in Janssens *et al.*[31].

To generate plasmids encoding lentiviral accessory proteins, HIV ORFs were subcloned into pcDNA4/TO (Invitrogen) carrying a 3' 2xStrepTagII-TEV-3xFLAG sequence as described in Jäger *et al.*[32].

Double-thymidine block of HeLa 'Kyoto' cells and propidium iodide (PI) staining

For synchronization, 0.5 x 10^6 HeLa 'Kyoto' cells were seeded per 10 cm-cell culture dish. Thymidine and 2'-deoxycytidine stock solutions (100 mM; both Sigma-Aldrich) were prepared in PBS and sterile filtered before use. 24 h after seeding, the medium was replaced with DMEM + 2 mM thymidine and cells were incubated for 16 h at 37 °C (= 1st block). Cells were washed 3 times with PBS and DMEM + 25 µM 2'-deoxycytidine was added for 8 h at 37 °C (= 1st release). Again, the medium was replaced with DMEM + 2 mM thymidine and cells were incubated for 16 h at 37 °C (= 2nd block). Subsequently, cells were washed 3 times with PBS and DMEM + 25 µM 2'-deoxycytidine was added (= 2nd release). Cells were harvested at different time points post-release and the respective samples split for immunoblot analysis and determination of cell cycle-phases by flow cytometry.

For the latter, cells were fixed with cold 70 % ethanol and incubated >2 h at 4 °C. After washing twice with PBS, cells were resuspended in 500 µL FxCycle PI/RNase Staining Solution (Life Technologies) and incubated for 30 min at room temperature (RT). Samples were analyzed using a BD Accuri C6 cytometer (BD Biosciences).

HIV-1 infection of synchronized HeLa cells or activated $CD4^+$ T cells and viral DNA quantitation byqPCR

For HIV-1 virion production, 2 x 10^7 HEK293T/17 cells per 175 cm^2-cell culture flask were seeded. Cells were co-transfected with proviral HIV-1 reporter plasmid mutated in p6 allowing for production of virions that package $SIVmac_{239}$ Vpx and lacking Vpr (pNL4.3 E^-R^- luc3 chp6; 8.75 µg)[33], an envelope vector for VSV-G-pseudotyping (pCMV-VSV-G; 4.375 µg) and an empty/ Vpx-expressing vector (pcDNA/ pcDNA6-$SIVmac_{239}$ Vpx myc-His; 4.375 µg) using 18 mM polyethylenimine (PEI) reagent (Sigma-Aldrich). Ca. 16 h post-transfection, cells were washed once with PBS and fresh DMEM added. 48 and 72 h post-transfection, cell culture supernatants were harvested, filtered (0.45 µm) and incubated with 1 U/ mL

DNaseI (NEB) for 1 h at RT. After incubation for 2 h on ice, viral stocks were purified through a 20 % sucrose cushion (2 h, 106750 g, 4 °C). Virion pellets were resuspended in PBS and stored at -80 °C. Viral stocks were titrated by serial dilution and β-galactosidase read-out on TZM-bl reporter cells.

3 x 10^4 HeLa ‘Kyoto’ cells/ 12-well were seeded and synchronized using a double-thymidine block. After the 2nd release, cells were counted at each time point to ensure the use of equal amounts of virus. Subsequently, cells were infected with a VSV-G-pseudotyped, full-length HIV-1 reporter viruses +/- Vpx lacking Vpr (pNL4.3 E^-R^- luc chp6_pcDNA, MOI 3; pNL4.3 E^-R^- luc chp6_Vpx, MOI 1.5). Prior to infection, used HIV-1 virus stocks were incubated with 10 U/ mL DNaseI (NEB) (30 min, 37 °C) to reduce contamination by plasmid DNA in subsequent qPCR assays. For control purposes, the used virus was heat-inactivated (65 °C, 20 min). Cells were spin occulated (30 min, 254 g, 32 °C) and the virus removed 1 h post-infection. In order to monitor cell cycle-phases/ SAMHD1 phosphorylation states, samples for immunoblotting were harvested at the time of infection and DNA harvest.

2 x 10^5 activated $CD4^+$ T cells/ 96-well were seeded and infected with a VSV-G-pseudotyped, full-length HIV-1 reporter viruses +/- Vpx lacking Vpr (pNL4.3 E^-R^- luc chp6_pcDNA and pNL4.3 E^-R^- luc chp6_Vpx, MOI 7.5 each). Prior to infection, used HIV-1 virus stocks were incubated with 10 U/ mL DNaseI (NEB) (30 min, 37 °C) to reduce contamination by plasmid DNA in subsequent qPCR assays. For control purposes, the used virus was heat-inactivated (95 °C, 10 min). Cells were spin occulated (1.5 h, 800 g, 32 °C) and the virus removed 2 h post-infection. With removal of virus, cells were arrested using 100 ng/ mL nocodazole (Sigma-Aldrich) for 16 h (= inf) or 24 h (= inf +N) at 37 °C. In order to allow cell cycle-progression from mitosis into G_1 phase, arrested $CD4^+$ T cells were washed twice with warm medium to remove nocodazole (after 16 h) and again incubated for 6 h at 37 °C (= inf). In order to monitor cell cycle-phases/ SAMHD1 phosphorylation states, samples for immunoblotting were harvested prior to infection and 24 h post-infection (= DNA harvest).

Total DNA was isolated 4 h post-infection (HeLa) or 24 h post-infection ($CD4^+$ T cells) using the DNeasy Blood & Tissue Kit (QIAGEN). The following primer-probe set was used to measure early RT products: Ert2f (5’-GTGCCCGTCTGTTGTGTGAC), Ert2r (5’-GGCGCCACTGCTAGAGATTT) and as probe ERT2

(5′-(FAM)-CTAGAGATCCCTCAGACCCTTTTAGTCAGTGTGG-(TAMRA)-3′). The following primer-probe set was used to measure late RT products: MH531 (5'-TGTGTGCCCGTCTGTTGTGT), MH532V (5'-GAGTCCTGCGTCGAGAGATC) and as probe LRT-P (5′-(FAM)-CAGTGGCGCCCGAACAGGGA-(TAMRA)-3′)[34,35]. To normalize the amount of DNA for each condition, the copy number of the cellular gene porphobilinogen deaminase (PBGD) was quantified using the following primer-probe set: PBGD1 (5'-AAGGGATTCACTCAGGCTCTTTC), PBGD2 (5'-GGCATGTTCAAGCTCCTTGG) and as probe PBGD-P (5'-[VIC]-CCGGCAGATTGGAGAGAAAAGCCTGT-[MGBNFQ]). Standard curves were run with each experiment, using plasmids containing the PBGD amplicon sequence or pNL4.3 E^{-}_luc3. The PCR conditions were as follows: initial denaturation/ heat activation 94 °C for 10 min, followed by 45 cycles of denaturation for 15 sec at 95 °C and annealing/ elongation for 1 min at 56 °C. qPCR assays were performed in a 384-well format (20 ng DNA/ reaction) using the 2X FastStart Universal Probe Master (Rox) (Roche) on an ABI7900 cycler (Applied Biosystems). Each sample was measured in technical triplicates.

Chemically induced mitotic exit assay

For the chemically induced mitotic exit assay[15], 4-5 x 10^5 HeLa 'Kyoto' cells were seeded in 5 mL DMEM/ 6 cm-cell culture dish. On day 1 (= 52 h before harvest), HeLa 'Kyoto' cells were transfected with siRNAs specific for PP2A B55α subunit or control siRNA (see section RNAi).

On day 2, HeLa 'Kyoto' cells were arrested using nocodazole (100 ng/ mL; Sigma-Aldrich) 17 h before chemical induction of mitotic exit. Directly before adding flavopiridol, arrested cells were incubated for 30 min in DMEM containing 30 µM MG-132 (Calbiochem) and collected by mitotic shake-off. After washing once with PBS (containing 30 µM MG-132), cells were resuspended in 800 µL PBS (containing 30 µM MG-132) and divided into 100 µL aliquots. Subsequently, cells aliquots were incubated at 37 °C and forced to exit mitosis by addition of flavopiridol (final concentration: 20 µM; Sigma-Aldrich). Single cell aliquots were lysed every 3 min (over a total time period of 18 min) using 20-40 µL radioimmunoprecipitation buffer (RIPA; 2 mM EDTA, 1 % (v/v) glycerol,137 mM NaCl, 1 % (v/v) NP40, 0.1 % (w/v) SDS, 0.5 % (w/v) sodium deoxycholate, 25 mM Tris [pH 8.0]), which was supplemented with cOmplete, Mini Protease Inhibitor Tablets (Roche) and PhosSTOP Tablets (Roche), and directly frozen at

-20 °C. Protein concentrations were determined based on the method of Bradford[36] using the Protein Assay Dye Reagent Concentrate (Bio-Rad) and 10 µg of total protein were used for immunoblotting.

dNTP quantification by single-nucleotide incorporation assay

For dATP quantitation during cell cycle-progression, 0.5 x 10^6 HeLa 'Kyoto' cells were initially seeded per 10 cm-cell culture dish and synchronized using a double-thymidine block. For each time point, cells harvested from two dishes were pooled and subsequently divided for dNTP measurement/ immunoblotting/ PI-staining. For dATP quantitation during mitotic exit, 4 x 10^5 HeLa 'Kyoto' cells were seeded in per 6 cm-cell culture dish. Cells harvested from 5 dishes were pooled and subsequently divided for dNTP measurements/ immunoblotting. For quantitation of all four dNTPs synchronized HeLa cells or primary cells (MDMs and resting $CD4^+$ T cells), 2 x 10^6 cells (for each cell cycle-phase in HeLa cells or each donor in primary cells) were harvested and lysed for dNTP measurements. Remaining cells were used for immunoblotting. To calculate the average of intracellular dNTP concentrations, previously published cell volumes were used (HeLa cells: 2349-3857 $µm^3$; MDMs: 2660 $µm^3$; resting $CD4^+$ T cells: 186 $µm^3$)[37,38].

Briefly, cell pellets were resuspended in 65 % methanol, vortexed for 2 min and incubated at 95 °C for 3 min. After centrifugation (3 min, 17000 g, RT), supernatants were transferred to fresh tubes and dried using a vacuum concentrator (Epppendorf). dATP levels were quantified by single-nucleotide incorporation assay[37] and normalized to the protein content of the respective sample.

RNA interference (RNAi)

For immunoblot analysis, 2 x 10^4 HeLa 'Kyoto' cells were seeded in 500 µL DMEM/ 24-well and incubated overnight at 37 °C. HeLa 'Kyoto' cells were then transfected with small interfering RNAs (siRNAs) specific for different isoforms of PP2A subunits or a scrambled control (see RNAi sequences listed below; QIAGEN) using Lipofectamine RNAiMAX Reagent (Invitrogen). Per 24-well, in brief, 1.5 µL RNAiMAX and 5 pmol siRNA (in total) were diluted in 25 µL Opti-MEM (Gibco) each. After combining the mixes and incubation for 5 min at RT, 50 µL siRNA-lipid complexes were directly added to the cells. 52 h post-transfection, cells were harvested for immunoblot analysis.

For the chemically induced mitotic exit assay[15], 4-5 x 10^5 HeLa 'Kyoto' cells were seeded in 5 mL DMEM/ 6 cm-cell culture dish and incubated overnight at 37 °C. 52 h before chemical induction of mitotic exit, HeLa 'Kyoto' cells were transfected as described above (siRNA amount/ dish: 50 pmol).

For siRNA-transfection of MDMs, 3 x 10^5 MDMs/ 24-well were seeded in 500 µL RPMI-1640 (supplemented with 10 % FBS, 2 mM L-glutamine, 10 mM HEPES, 1 mM sodium pyruvate) and allowed to re-attach for 2 h at 37 °C. MDMs were transfected with siRNAs specific to PP2A B55α subunit, PP2A Cα subunit or a scrambled control using Stemfect RNA Transfection Kit (Stemgent). Per 24-well, 0.52 µL Stemfect reagent and 10 pmol siRNA (in total) were diluted in 12.5 µL Stemfect buffer each. After combining the mixes and incubation for 15 min at RT, 25 µL siRNA-lipid complexes were added to the MDMs. After 24 h, the medium was changed and MDMs were again transfected with siRNAs as described above. 48 h or up to 5 days post-transfection, MDMs were harvested for immunoblot and RT-qPCR analysis.

GeneID	Gene symbol	HGNC name	mRNA accession	siRNA target sequence	Product ID	Product name
5515	*PPP2CA*	protein phosphatase 2, catalytic subunit, alpha isozyme	NM_002715	ATGGAACTTGACGATACTCTA	SI02225783	Hs_PPP2CA_5
5518	*PPP2R1A*	protein phosphatase 2 regulatory subunit A, alpha	NM_014225	GACCAGGATGTGGACGTCAAA	SI04436495	Hs_PPP2R1A_7
5520	*PPP2R2A*	protein phosphatase 2 regulatory subunit B, alpha	NM_002717	CTGCAGATGATTTGCGGATTA	SI02228525	Hs_PPP2R2A_5
-	-	-	-	-	1027310	Negative Control

Identification of SAMHD1 interaction partners using mass spectrometry (MS)

MS analysis (in-gel digestion) using CBP-SBP-SAMHD1 in HEK293T cells

For tandem affinity purification (TAP), 1.5 x 10^7 HEK293T cells/ 175 cm^2 cell culture flask were seeded 16 h prior to transfection. The medium was removed and substituted by 10 mL DMEM (containing 15 % FBS). Cells were transfected with 35 µg total plasmid DNA (pNTAPB-SAMHD1) per flask using 18 mM PEI reagent (Sigma-Aldrich). 6 h post-transfection, the medium was replaced with 20 mL fresh DMEM. To purify sufficient amounts of protein complexes, a total of 1.5 x 10^8 cells were subjected to TAP using the InterPlay TAP Purification Kit (Agilent Technologies) according to the manufacturer's instructions.

To reduce the volume of the final protein eluate, trichloroacetic acid (TCA) precipitation was performed. In brief, 80 % TCA solution was added to the sample to a final concentration of 4 %. The sample was incubated for 30 min on ice and then centrifuged at 15000 g for 10 min, 4 °C. Since the precipitated proteins formed a solid pellet, the supernatant was discarded. To wash away residual TCA, 500 µL 80 % acetone were added and the sample thoroughly vortexed. Again, the sample was centrifuged (15000 g, 10 min, 4 °C) and the supernatant discarded. The acetone wash step was repeated four additional times. Finally, the protein pellet was dried for 30 min and resuspended in 50 µL 4X Laemmli sample buffer (50 % (v/v) glycerol, 5 % (w/v) SDS, 31.25 % (v/v) stacking gel buffer, without dye). The proteins were resuspended at 4 °C overnight under constant rotation. The protein concentration was determined via BCA protein assay (Pierce Biotechnology). Protein samples were prepared by adding 4X Laemmli sample buffer, mixed with 20 % (v/v) β-mercaptoenthanol (Sigma-Aldrich), to a final 1X concentration and denatured at 95 °C for 5 min. 20 µg total protein were subjected to SDS-PAGE (10 %) and gels were run at 20 mA/ gel in 1X SDS running buffer (1.9 M glycine, 1 % (w/v) SDS, 0.25 M Tris).

For subsequent MS analysis, gels were stained overnight with Coomassie Brilliant Blue G-250 (Bio-Rad) at RT under constant agitation using Coomassie staining solution (7 % (v/v) acetic acid, 0.5 % (w/v) Coomassie Brilliant Blue G-250, 40 % (v/v) methanol). Gels were destained with several changes of Coomassie destaining solution (7 % (v/v) acetic acid, 40 % (v/v) methanol), until protein bands were clearly visible. Visible protein bands were excised, in-gel digested using trypsin and analyzed by mass spectrometry (nano-UPL nanoESI-MS^E) as published previously[39]. Differing to this study, MS^E data were

searched against a UniProt database restricted to reviewed entries of *Homo sapiens* (taxon identifier: 9606).

Additionally, gradual purification of SAMHD1-associated protein complexes during TAP was monitored. Samples of intermediate steps (20 µL each) were separated using a 4-12 % NuPAGE Bis-Tris gradient gels (Life Technologies) and stained with SYPRO Ruby Protein Gel Stain (Life Technologies) after electrophoresis - according to the manufacturer's instructions. Briefly, gels were placed in 100 mL fix solution (7 % (v/v) acetic acid, 50 % (v/v) methanol) and agitated for 30 min twice. Afterwards, 60 mL SYPRO Ruby gel stain were added and the gel agitated overnight. After washing with 100 mL wash solution (7 % (v/v) acetic acid, 10 % (v/v) methanol) for 30 min, protein bands were detected using a gel documentation system (Intas Science Imaging).

MS analysis (on-bead digestion) using GFP-SAMHD1 in HEK293T cells

For GFP-trapping, 3 x 10^6 HEK293T cells/ 10 cm-cell culture dish were seeded. After 24 h, HEK293T cells were transfected with 6 µg (pEGFP-C1-SAMHD1 or pEGFP-C1) of total plasmid DNA per dish using 18 mM PEI reagent (Sigma-Aldrich). 48 h post-transfection, cells were harvested, pooled and lysed in 1 mL NET lysis buffer/ sample.

Anti-GFP trapping of the same amount of lysates from GFP or GFP-SAMHD1 overexpressing cells was executed; notably, GFP-trapped complexes were washed stringently for four times using NENT300 (20 mM Tris [pH 7.4], 300 mM NaCl, 1 mM EDTA [pH 7.4], 0.1 % NP40, 25 % glycerol). GFP-traps were subjected to on-bead trypsin digestion and the resulting peptide mixture was analyzed by LC-MS/MS on a nano-LC hybrid quadrupole-orbitrap mass spectrometer (Q Exactive, Thermo Fisher Scientific). Relative quantification of proteins in different conditions was executed with Progenesis software (Nonlinear Dynamics) incorporating protein identifications obtained by MASCOT (Matrix Science) search engine using Swiss-Prot (*Homo sapiens*, 20,202 entries) as a database. Only peptides with 95 % peptide identification probability, resulting in a 1 % peptide FDR (Scaffold), were taken into account during the analysis. As a result, a list of proteins present in the GFP-SAMHD1 trap and not present in the GFP trap could be established (280 hits). CDK1 and the PP2A Aα (= 65 kDa) subunit were present in this list.

Co-immunoprecipitation (CoIP)

With transfected nFLAG-SAMHD1 in HEK293T cells

For CoIPs, 3 x 10^6 HEK293T cells/ 10 cm-cell culture dish were seeded. After 24 h, HEK293T cells were transfected with 6 µg (pcDNA3.1(+)-nFLAG-SAMHD1 only) or 11 µg (5.5 µg pcDNA3.1(+)-nFLAG-SAMHD1 + 5.5 µg PP2A B-type subunits in pEGFP (= GFP-tagged)) of total plasmid DNA per dish using 18 mM PEI reagent (Sigma-Aldrich). 48 h post-transfection, cells were harvested and lysed in 200 µL NET lysis buffer (50 mM Tris/ HCl [pH 7.4], 150 mM NaCl, 15 mM EDTA [pH 7.4], 1 % NP40 containing cOmplete Protease Inhibitor Cocktail [Roche] and PhosSTOP Phosphatase Inhibitor Cocktail [Roche]) per dish for 30 min on ice. Lysates were centrifuged at 17000 g for 15 min at 4 °C. For later analysis of the input, 25 µL lysate were directly diluted in 25 µL 2X NuPAGE LDS Sample Buffer (Life Technologies), boiled (95 °C, 5 min) and stored at -20 °C. The remaining lysate was pre-cleared using 25 µL Protein G Sepharose 4 Fast Flow (GE Healthcare) in 500 µL TBS + 0.1 % NP40 (containing cOmplete Protease Inhibitor Cocktail [Roche]) for 1.5 h at 4 °C. After centrifugation, 200 µL NENT100 + 1 mg/ mL BSA (20 mM Tris [pH 7.4], 100 mM NaCl, 1 mM EDTA [pH 7.4], 0.1 % NP40, 25 % glycerol) was added to the pre-cleared lysate, which then was incubated with 25 µL ANTI-FLAG M2 Affinity Gel (Sigma-Aldrich) for 1 h at 4 °C. Subsequently, beads were washed twice with 300 µL NENT300 (20 mM Tris [pH 7.4], 300 mM NaCl, 1 mM EDTA [pH 7.4], 0.1 % NP40, 25 % glycerol) and twice with 300 µL TBS + 0.1 % NP40, each time for 2 min at 4 °C under constant rotation. After centrifugation, the supernatant was removed and bound immune complexes were eluted in 25 µL 2X NuPAGE LDS Sample Buffer through boiling (95 °C, 5 min).

With transfected GST-tagged PP2A B subunits in HEK293T cells

For CoIPs, 3 x 10^6 HEK293T cells/ 10 cm-cell culture dish were seeded. After 24 h, HEK293T cells were transfected with 11 µg of total plasmid DNA (3.5 µg pcDNA3.1(+)-nFLAG-SAMHD1 + 7.5 µg PP2A B-type subunits in pGMEX-T1 (= GST-tagged)) per dish using 18 mM PEI reagent (Sigma-Aldrich). 48 h post-transfection, cells were harvested and lysed in 200 µL NET lysis buffer (containing cOmplete Protease Inhibitor Cocktail [Roche] and PhosSTOP Phosphatase Inhibitor Cocktail [Roche]) per dish for 30 min on ice. Lysates were centrifuged at 17000 g for 15 min at 4 °C. For later analysis of the input, 25 µL lysate were directly diluted in 25 µL 2X NuPAGE LDS Sample Buffer (Life Technologies), boiled (95 °C, 5 min)

and stored at -20 °C. 25 µL GST-Trap_M beads (Chromotek)/ sample were washed twice with ice-cold dilution/ wash buffer (10 mM Tris-HCl [pH 7.5], 150 mM NaCl, 0.5 mM EDTA) and incubated together with 175 µL lysate as well as 500 µL dilution/ wash buffer (containing cOmplete Protease Inhibitor Cocktail [Roche] and 1 mg/ mL BSA) for 1.5 h at 4 °C. Subsequently, beads were washed twice with 500 µL dilution/ wash buffer and twice with 500 µL dilution/ wash buffer 2 (10 mM Tris-HCl [pH 7.5], 500 mM NaCl, 0.5 mM EDTA), each time for 2 min at 4 °C under constant rotation. After separating the beads magnetically, the supernatant was removed and bound immune complexes were eluted in 25 µL 2X NuPAGE LDS Sample Buffer through boiling (95 °C, 5 min).

With endogenous SAMHD1 in cycling THP-1 cells

For endogenous CoIPs, 2 x 10^7 cycling THP-1 cells per condition were harvested and lysed in 200 µL NET lysis buffer (containing cOmplete Protease Inhibitor Cocktail [Roche] and PhosSTOP Phosphatase Inhibitor Cocktail [Roche]) each for 30 min on ice. Lysates were centrifuged at 17000 g for 15 min at 4 °C. For later analysis of the input, 25 µL lysate were directly diluted in 25 µL 2X NuPAGE LDS Sample Buffer (Life Technologies), boiled (95 °C, 5 min) and stored at -20 °C. The remaining lysate was pre-cleared using 50 µL Protein G Sepharose 4 Fast Flow (GE Healthcare) and 2.5 µg anti-mouse (G3A1) mAb IgG1 isotype control (CST) in 500 µL TBS + 0.1 % NP40 (containing cOmplete Protease Inhibitor Cocktail [Roche]) for 1.5 h at 4 °C. After centrifugation, the pre-cleared lysate was transferred to a new tube and was then incubated with 5 µg anti-SAMHD1 (antibodies-online) or anti-mouse (G3A1) mAb IgG1 isotype control (CST). After 2 h at 4 °C, 50 µL Protein G Sepharose 4 Fast Flow (GE Healthcare) were added to each sample for 1.5 h at 4 °C. Subsequently, beads were washed twice with 300 µL NENT300 and twice with 300 µL TBS + 0.1 % NP40, each time for 2 min at 4 °C under constant rotation. After centrifugation, the supernatant was removed and bound immune complexes were eluted in 25 µL 2X NuPAGE LDS Sample Buffer through boiling (95 °C, 5 min).

Immunoblotting

For immunoblotting, cells were harvested and lysed in RIPA buffer (supplemented with protease and phosphatase inhibitor cocktails) for 30 min on ice. After centrifugation (30 min, 17000 g, 4 °C), protein

concentrations were determined, based on the method of Bradford[36] using the Protein Assay Dye Reagent Concentrate (Bio-Rad).

Protein samples were prepared by adding NuPAGE LDS Sample Buffer (4X) and NuPAGE Sample Reducing Agent (10X), containing dithiothreitol (DTT), to a final 1X concentration each. Proteins were denatured at 70 °C for 10 min. Pre-casted 4-12 % NuPAGE Bis-Tris gradient gels (Life Technologies) were run in 1X MOPS running buffer (1 M MOPS, 1 M Tris, 69.3 mM SDS, 20.5 mM EDTA Titriplex II) at 200 V. Separated proteins were transferred to a Hybond P 0.45 PVDF membrane (GE Healthcare), using the XCell II Blot Module and 1X NuPAGE Transfer Buffer (Life Technologies). Membranes were blocked in 0.01 % (v/v) Tris-buffered saline with Tween 20 (TBST) + 5 % (w/v) powdered milk or BSA (both Carl Roth) for 2 h at 4 °C. Primary antibodies were applied overnight at 4 °C. As secondary antibodies, horseradish peroxidase (HRP)-linked $F(ab')_2$ fragments (GE Healthcare) or anti-mouse/ rabbit IgG, HRP-linked antibodies (Cell Signaling Technology (CST)) were used (for 2 h, 4 °C). For detection, ECL Prime reagent (GE Healthcare) and Lumi-Light Western Blotting Substrate (Roche) were employed. The emitted chemiluminescence was detected at different exposure times using autoradiography films (Fujifilm). For removal of antibodies, probed membranes were rotated for 30-60 min at 65 °C in Stripping buffer (2 % (w/v) SDS, 62.5 mM Tris-HCl [pH 6.8], 100 mM β-mercaptoethanol).

Detection of proteins was performed with the following antibodies: anti-cdc2/ CDK1 (#9116; 1:1000), anti-CDK2 (#2546; 1:1000), anti-Cyclin A2 (#4656; 1:500 – 1:1000), anti-Cyclin A2 (18202-1-AP, Proteintech; 1:1000), anti-Cyclin B1 (#4138; 1:1000), anti-FLAG M2 (F3165, Sigma-Aldrich; 1:500 – 1:5000), anti-GAPDH (#2118; 1:1000 – 1:5000), anti-GFP (#2956; 1:1000 – 1:5000), anti-GST (26H1) (#2624; 1:2000 – 1:5000), anti-mouse (G3A1) mAb IgG1 isotype control (#5415; 5 µg for CoIPs), anti-phospho-CDK substrate [pTPXK] (#14371; 1:2000), anti-PP2A A subunit (#2039; 1:500 – 1:1000), anti-PP2A B subunit (#4953; 1:500 – 1:1000), anti-PP2A B56δ subunit (HPA029046, Sigma-Aldrich; 1:1000), anti-PP2A C subunit (#2259; 1:500 – 1:1000), monoclonal anti-PP2A C subunit (clone F2.5G4, kind gift from Stephen Dilworth, Middlesex University London, UK; 1:1000), anti-PRC1 (#3639; 1:1000), anti-PRC1 (phospho T481) [EP1514Y] (ab62366, Abcam; 1:1000), anti-SAMHD1 (ABIN2453936, antibodies-online; 5 µg for CoIPs), anti-SAMHD1 (12586-1-AP, Proteintech; 1:1000 – 1:2000), anti-SAMHD1 pT592 (#15038; 1:1000), anti-SAMHD1 pT592 (kind gift from Oliver T. Keppler, LMU Munich, Germany; 1:1000), anti-SAMHD1 pT592 (PA5-38029, Thermo Fisher Scientific; 1:1000), anti-STAT1 (sc-346, Santa Cruz

Biotechnology; 1:5000). If not stated otherwise, antibodies were purchased from Cell Signaling Technology (CST).

Quantification of immunoblot data was carried out using Image Studio Lite 5.0.21 software (LI-COR). Uncropped scans of immunoblots are supplied in the Supplementary Information.

***In vitro*-phosphorylation using CDK1/ cyclin B1**

Recombinant SAMHD1 purified from Sf9 cells[40] was incubated with 0.02 µg/ µL recombinant CDK1/ cyclin B1 complex (ProQinase) at 30 °C for up to 2 h. The used reaction buffer contained 60 mM HEPES-NaOH [pH 7.5], 3 mM $MgCl_2$, 3 mM $MnCl_2$, 1.2 mM DTT, 50 µg/ mL PEG20,000, 1 mM cold ATP and 3 µM Na-orthovanadate. Subsequently, reaction mixtures were analyzed by immunoblotting.

***In vitro*-PP2A dephosphorylation assays and phospho-MS analysis**

De novo purified $PP2A_D$ and PP1 were obtained from a commercial source (Millipore). For comparative MS analysis, 1 µg of recombinant SAMHD1 was incubated in enzyme dilution buffer (0.15 M NaCl, 20 mM MOPS, pH 7.5, 60 mM 2-mercaptoethanol, 0.1 mM $MnCl_2$, 1 mM $MgCl_2$, 1 mM EGTA, 10 % glycerol and 0.1 mg/ mL serum albumin) with 0.032 units/ µL PP2A, 0.032 units/ µL PP1 or buffer only, for 1 h at 30 °C, in a reaction volume of 10 µL. 1 µL of complete protease inhibitor mix (Roche) was also included. Equilibrated units of $PP2A_D$ and PP1 were used in all experiments (one unit released 1 nmol of phosphate/ min from 15 µM phosphorylase-*a* at 30 °C, a well-known substrate of both PP1 and PP2A). Reactions were stopped by adding 5x SDS-PAGE sample buffer and 10 min of boiling. Following SDS-PAGE of the samples, the 1D-gel piece containing SAMHD1 was subjected to in-gel trypsin digestion followed by C18 peptide cleanup (ZipTip, Millipore). The resulting peptide mixture was subjected to targeted LC-MS/MS on a nano-LC hybrid quadrupole-orbitrap mass spectrometer (Q Exactive, Thermo Fisher Scientific). Data analysis was executed with Pinpoint™ 1.4.0 (Thermo Fisher Scientific) software. Normalization of data in the different conditions was done based on a non-phosphorylatable peptide of SAMHD1 (GGFEEPVLLK) (see Supplementary Table 1). Calculation of phosphorylation stoichiometry was executed as described in Schreurs *et al.*[41], and based on a method described by Olsen *et al.*[42]. For immunoblot analysis with anti-SAMHD1 pT592 antibodies, the amount of SAMHD1 was reduced to 0.1 µg per dephosphorylation reaction. For *in vitro*-dephosphorylation with PP2A trimers, the relevant N-

terminally GFP-tagged B-type subunits (B55α, B56α, B56β, PR72) were first expressed in HEK293T cells, and the respective catalytically competent PP2A trimers harboring a specific B-type subunit were retrieved from the transfected cells by GFP-trapping, as previously described[43]. Whenever OA (Calbiochem) was used in the assays, the phosphatases were first pre-incubated with 50 nM OA for 10 min at 30 °C, before their addition to the substrate. All dephosphorylation reactions were stopped by adding 5x SDS-PAGE sample buffer and boiling.

Computer programs

Representations of SAMHD1 structure were generated using USCF Chimera[44] based on published coordinates (PDB-ID: 4BZC[19] and 4CC9[20]).

Supplementary References

1. Barr, F. A., Elliott, P. R. & Gruneberg, U. Protein phosphatases and the regulation of mitosis. *J. Cell Sci.* **124,** 2323–2334 (2011).
2. Gharbi-Ayachi, A. *et al.* The substrate of Greatwall kinase, Arpp19, controls mitosis by inhibiting protein phosphatase 2A. *Science* **330,** 1673–1677 (2010).
3. Mochida, S., Maslen, S. L., Skehel, M. & Hunt, T. Greatwall phosphorylates an inhibitor of protein phosphatase 2A that is essential for mitosis. *Science* **330,** 1670–1673 (2010).
4. Rogers, S. *et al.* PP1 initiates the dephosphorylation of MASTL, triggering mitotic exit and bistability in human cells. *J. Cell Sci.* **129,** 1340–1354 (2016).
5. Ren, D. *et al.* Cell cycle-dependent regulation of Greatwall kinase by protein phosphatase 1 and regulatory subunit 3B. *J. Biol. Chem.* **292,** 10026–10034 (2017).
6. Heim, A., Konietzny, A. & Mayer, T. U. Protein phosphatase 1 is essential for Greatwall inactivation at mitotic exit. *EMBO Rep.* **16,** 1501–1510 (2015).
7. Ma, S. *et al.* Greatwall dephosphorylation and inactivation upon mitotic exit is triggered by PP1. *J. Cell Sci.* **129,** 1329–1339 (2016).
8. Williams, B. C. *et al.* Greatwall-phosphorylated Endosulfine is both an inhibitor and a substrate of PP2A-B55 heterotrimers. *eLife* **3,** e01695 (2014).
9. Hégarat, N. *et al.* PP2A/B55 and Fcp1 regulate Greatwall and Ensa dephosphorylation during mitotic exit. *PLoS Genet.* **10,** e1004004 (2014).
10. Della Monica, R., Visconti, R., Cervone, N., Serpico, A. F. & Grieco, D. Fcp1 phosphatase controls Greatwall kinase to promote PP2A-B55 activation and mitotic progression. *eLife* **4** (2015).
11. Filter, J. J., Williams, B. C., Eto, M., Shalloway, D. & Goldberg, M. L. Unfair competition governs the interaction of pCPI-17 with myosin phosphatase (PP1-MYPT1). *eLife* **6** (2017).
12. St Gelais, C. *et al.* Identification of cellular proteins interacting with the retroviral restriction factor SAMHD1. *J. Virol.* **88,** 5834–5844 (2014).
13. White, T. E. *et al.* The retroviral restriction ability of SAMHD1, but not its deoxynucleotide triphosphohydrolase activity, is regulated by phosphorylation. *Cell Host Microbe* **13,** 441–451 (2013).
14. Cribier, A., Descours, B., Valadao, A. L. C., Laguette, N. & Benkirane, M. Phosphorylation of SAMHD1 by cyclin A2/CDK1 regulates its restriction activity toward HIV-1. *Cell Rep.* **3,** 1036–1043 (2013).
15. Schmitz, M. H. A. *et al.* Live-cell imaging RNAi screen identifies PP2A-B55alpha and importin-beta1 as key mitotic exit regulators in human cells. *Nat. Cell Biol.* **12,** 886–893 (2010).

16. Ahn, J. *et al.* HIV/simian immunodeficiency virus (SIV) accessory virulence factor Vpx loads the host cell restriction factor SAMHD1 onto the E3 ubiquitin ligase complex CRL4DCAF1. *J. Biol. Chem.* **287,** 12550–12558 (2012).

17. Daddacha, W. *et al.* SAMHD1 Promotes DNA End Resection to Facilitate DNA Repair by Homologous Recombination. *Cell Rep.* **20,** 1921–1935 (2017).

18. St Gelais, C. *et al.* A Putative Cyclin-binding Motif in Human SAMHD1 Contributes to Protein Phosphorylation, Localization, and Stability. *J. Biol. Chem.* **291,** 26332–26342 (2016).

19. Ji, X. *et al.* Mechanism of allosteric activation of SAMHD1 by dGTP. *Nat. Struct. Mol. Biol.* **20,** 1304–1309 (2013).

20. Schwefel, D. *et al.* Structural basis of lentiviral subversion of a cellular protein degradation pathway. *Nature* **505,** 234–238 (2014).

21. Mo, S.-T. *et al.* Visualization of subunit interactions and ternary complexes of protein phosphatase 2A in mammalian cells. *PloS one* **9,** e116074 (2014).

22. Lambrecht, C., Haesen, D., Sents, W., Ivanova, E. & Janssens, V. Structure, regulation, and pharmacological modulation of PP2A phosphatases. *Methods Mol. Biol.* **1053,** 283–305 (2013).

23. Ryoo, J. *et al.* The ribonuclease activity of SAMHD1 is required for HIV-1 restriction. *Nat. Med.* **20,** 936–941 (2014).

24. Baldauf, H.-M. *et al.* SAMHD1 restricts HIV-1 infection in resting CD4(+) T cells. *Nat. Med.* **18,** 1682–1687 (2012).

25. Long, L. *et al.* Recruitment of phosphatase PP2A by RACK1 adaptor protein deactivates transcription factor IRF3 and limits type I interferon signaling. *Immunity* **40,** 515–529 (2014).

26. Okamoto, K. *et al.* Cyclin G recruits PP2A to dephosphorylate Mdm2. *Mol. Cell* **9,** 761–771 (2002).

27. Yoh, S. M. *et al.* PQBP1 Is a Proximal Sensor of the cGAS-Dependent Innate Response to HIV-1. *Cell* **161,** 1293–1305 (2015).

28. Berger, A. *et al.* SAMHD1-deficient CD14+ cells from individuals with Aicardi-Goutieres syndrome are highly susceptible to HIV-1 infection. *PLoS Pathog.* **7,** e1002425 (2011).

29. Larkin, M. A. *et al.* Clustal W and Clustal X version 2.0. *Bioinformatics* **23,** 2947–2948 (2007).

30. Longin, S. *et al.* Selection of protein phosphatase 2A regulatory subunits is mediated by the C terminus of the catalytic Subunit. *J. Biol. Chem.* **282,** 26971–26980 (2007).

31. Janssens, V. *et al.* Identification and functional analysis of two Ca2+-binding EF-hand motifs in the B"/PR72 subunit of protein phosphatase 2A. *J. Biol. Chem.* **278,** 10697–10706 (2003).

32. Jäger, S. *et al.* Global landscape of HIV-human protein complexes. *Nature* **481,** 365–370 (2011).

33. Sunseri, N., O'Brien, M., Bhardwaj, N. & Landau, N. R. Human immunodeficiency virus type 1 modified to package Simian immunodeficiency virus Vpx efficiently infects macrophages and dendritic cells. *J. Virol.* **85,** 6263–6274 (2011).

34. Butler, S. L., Hansen, M. S. & Bushman, F. D. A quantitative assay for HIV DNA integration in vivo. *Nat. Med.* **7,** 631–634 (2001).

35. König, R. *et al.* Global analysis of host-pathogen interactions that regulate early-stage HIV-1 replication. *Cell* **135,** 49–60 (2008).

36. Bradford, M. M. A rapid and sensitive method for the quantitation of microgram quantities of protein utilizing the principle of protein-dye binding. *Anal. Biochem.* **72,** 248–254 (1976).

37. Diamond, T. L. *et al.* Macrophage tropism of HIV-1 depends on efficient cellular dNTP utilization by reverse transcriptase. *J. Biol. Chem.* **279,** 51545–51553 (2004).

38. Park, K. *et al.* 'Living cantilever arrays' for characterization of mass of single live cells in fluids. *Lab Chip* **8,** 1034–1041 (2008).

39. Spiric, J., Engin, A. M., Karas, M. & Reuter, A. Quality Control of Biomedicinal Allergen Products - Highly Complex Isoallergen Composition Challenges Standard MS Database Search and Requires Manual Data Analyses. *PloS one* **10,** e0142404 (2015).

40. White, T. E. *et al.* Contribution of SAM and HD domains to retroviral restriction mediated by human SAMHD1. *Virology* **436,** 81–90 (2013).

41. Schreurs, S. *et al.* In vitro phosphorylation does not influence the aggregation kinetics of WT alpha-synuclein in contrast to its phosphorylation mutants. *Int. J. Mol. Sci.* **15,** 1040–1067 (2014).

42. Olsen, J. V. *et al.* Quantitative phosphoproteomics reveals widespread full phosphorylation site occupancy during mitosis. *Sci. Signal.* **3,** ra3 (2010).

43. Haesen, D. *et al.* Recurrent PPP2R1A Mutations in Uterine Cancer Act through a Dominant-Negative Mechanism to Promote Malignant Cell Growth. *Cancer Res.* **76,** 5719–5731 (2016).

44. Pettersen, E. F. *et al.* UCSF Chimera - a visualization system for exploratory research and analysis. *J. Comput. Chem.* **25,** 1605–1612 (2004).
